# An Enhanced A*-DWA Fusion Algorithm for Robot Navigation in Complex Environments

**DOI:** 10.3390/biomimetics11020138

**Published:** 2026-02-12

**Authors:** Huifang Bao, Jie Fang, Mingxing Fang, Jinsi Zhang, Zhuo Zhang, Haoyu Cai

**Affiliations:** 1 School of Electrical and Photoelectronic Engineering, West Anhui University, Lu’an 237012, China; 42000010@wxc.edu.cn (H.B.); jinsizhang@wxc.edu.cn (J.Z.); 2025210016@mail.wxc.edu.cn (Z.Z.); 2023010462@mail.wxc.edu.cn (H.C.); 2 Anhui Undergrowth Crop Intelligent Equipment Engineering Research Center, Lu’an 237012, China; 3 Anhui Engineering Research Center on Information Fusion and Control of Intelligent Robot, Wuhu 241002, China; mxfang@ahnu.edu.cn

**Keywords:** mobile robot, enhanced A*, DWA, fusion planning, complex environment

## Abstract

To tackle the navigation challenge in dynamic and complex environments, this study designs a fusion planning framework that synergistically integrates enhanced A* algorithm with improved DWA, inspired by the biological dual-layer navigation mechanism of global path planning and local real-time obstacle avoidance. Firstly, the original global path from the conventional A* algorithm is smoothed and length-reduced through a three-stage optimization strategy involving redundant node removal and forward and reverse path relaxation, mimicking the behavioral logic of honeybees and desert ants that eliminate redundant routes to complete foraging and homing with minimal energy consumption. Secondly, an evaluation function integrating dynamic obstacle perception and adaptive weight adjustment is designed for the DWA to enhance the intelligence of local planning, drawing on the adaptive strategy of animals such as antelopes that adjust behavioral priorities according to environmental complexity to balance safety and efficiency. To comprehensively verify the performance of the proposed algorithm, simulation evaluations are performed in various scenarios, including 20 × 20 and 30 × 30 grid maps, with single and dual dynamic obstacles. Results demonstrate that our algorithm outperforms conventional methods in path length, smoothness, and safety. Further physical verification is carried out on a LiDAR-equipped mobile robot (Shenzhen Yuanchuangxing Technology Co., Ltd., Shenzhen, China) based on the ROS platform, confirming that the algorithm can stably achieve static path tracking and real-time obstacle avoidance in real indoor environments. Consequently, the developed hybrid algorithm delivers a viable and robust solution for autonomous mobile robots to navigate safely and efficiently in unpredictable and complex environments.

## 1. Introduction

For mobile robots to operate autonomously in complex settings, effective path planning is indispensable. It underpins their capacity for intelligent task execution and is a key factor driving their growing adoption across intelligent manufacturing, warehousing and logistics, outdoor inspection, and other related fields. Based on the degree of environmental information availability, path planning is generally categorized into global and local [1]. The former relies on known environmental map information and aims to obtain a theoretically optimal path; the latter relies on real-time sensor perception data and is primarily used for dynamic obstacle avoidance and trajectory tracking [2]. As the dynamics and complexity of the operating environment continue to increase, robots need to generate safe, smooth, and globally consistent motion trajectories in real time within mixed scenarios that include static structures and dynamic disturbances. In such cases, traditional single planning methods often struggle to simultaneously meet multiple requirements such as safety, optimality, smoothness, and real-time performance [3]. Therefore, integrating the guiding capability of global planning with the responsive capability of local planning to construct an efficient and collaborative hybrid path planning algorithm has become an important research direction for enhancing the navigation performance of mobile robots in dynamic environments, and is of great significance for advancing their practical engineering applications.

From a biomimetic perspective, the navigation mechanisms of natural organisms offer profound insights for addressing the aforementioned challenges [4]. In nature, numerous organisms have evolved sophisticated capabilities for efficient navigation and robust obstacle avoidance in complex environments. For instance, honeybees communicate food locations and plan optimal flight paths via their “dance language” [5]; desert ants leverage celestial cues and visual feedback to synergistically achieve global navigation and local obstacle avoidance [6]; and migratory birds exemplify a seamless integration of macroscale route planning and microscale dynamic adjustments [7]. The core commonality of these biological intelligences lies in an efficient collaborative mechanism of “global path optimization and local real-time response”. Inspired by this, this study aims to draw on this bionics-inspired decision-making logic to construct a novel hybrid path planning framework in order to enhance the autonomous navigation performance of mobile robots in dynamic and cluttered environments.

In global path planning, the A* algorithm serves as a widely used tool for generating robot trajectories, attributed to its efficient heuristic search mechanism and theoretical completeness [8]. However, the original A* algorithm has significant limitations: it does not consider the physical dimensions of the robot, and the generated paths contain numerous redundant nodes and sharp turning points, which not only increase the path length but also requires the robot to frequently start, stop, and turn, making it difficult to directly apply it to actual motion control. To address these issues, scholars have conducted targeted research: Hart et al. [9] proposed the basic framework of the A* algorithm and verified its effectiveness in path search, but their research was based on an ideal discrete grid space, making it difficult for the generated path to be directly tracked and executed by an actual robot. Duchoň et al. [10] adopted the Jump Point Search (JPS) method, which achieved significant improvement in computational efficiency by identifying jump points and pruning redundant neighboring nodes; however, this mechanism omits the search of part of the space, resulting in a suboptimal final path length. Fu et al. [11] enhanced search efficiency in the A* algorithm and reduced path turns through multi-neighborhood hybrid search and node optimization strategies; yet, this method relies on an oversimplified point-mass model of the robot, failing to account for its physical dimensions, resulting in the planned path lacking a safety margin when approaching obstacles and posing potential collision risks. Wang et al. [12] ensured a safe distance from static obstacles by expanding the robot’s radius around them; nevertheless, this operation reduces the navigable area, making the path prone to excessive detours in dense obstacle scenarios. Bhadoria et al. [13] filtered optimal nodes by introducing a target direction angle factor into the heuristic function and eliminated redundant expanded nodes, but they did not perform path smoothing, which renders the motion of the mobile robot neither smooth nor efficient. Zhang et al. [14] applied cubic quasi-uniform B-spline curves for smoothing the path obtained from their enhanced A* algorithm; although this reduces path inflection points and improves the navigation adaptability of Unmanned Surface Vehicles (USVs), it did not fully consider the hull kinematic parameters of USVs; thus, limiting its ability to fulfill the operational demands of complex real-world waters.

For local, real-time planning tasks, the Dynamic Window Approach (DWA) has become one of the mainstream local obstacle avoidance algorithms due to its ability to fully consider the kinematic constraints of robots and perform real-time speed sampling [15]. However, the traditional DWA still exhibits numerous limitations in dynamic and complex environments: for instance, its initial heading angle is often set to a fixed value or only points to the final goal, which can easily lead to circuitous trajectories in the initial phase of complex scenarios; the weights of the evaluation function are mostly set based on experience and cannot be adaptively adjusted according to the degree of environmental congestion, resulting in limited responsiveness to dynamic obstacles; and it tends to converge to local optima, or suffer from complete deadlock in narrow unstructured regions, which restricts its overall performance in dynamic multi-obstacle environments. To overcome these limitations, scholars have enhanced and improved the DWA algorithm from various perspectives. Kim et al. [16] integrated deep reinforcement learning (DRL) with a dedicated velocity control module to dynamically adjust the linear and angular velocity output by the DWA, significantly improving the local dynamic obstacle avoidance capability of mobile robots in complex scenarios. Wang et al. [17] proposed an improved DWA that incorporates the influences of ocean waves and currents, enabling real-time local trajectory generation for Unmanned Surface Vehicles (USVs). Xu et al. [18] incorporated road guidelines generated by B-spline curve fitting into the evaluation function to optimize the local path decision-making of the DWA, remarkably improving the path conformity and navigation efficiency of epidemic prevention robots in outdoor road environments. Nevertheless, although these improved measures have enhanced local obstacle avoidance performance, in the absence of macro-level route guidance, they are liable to result in local trajectories deviating from the direction of optimal global path, thus leading to detours and sacrificing overall path efficiency.

To synthesize the requirements of global optimality and local real-time performance [19], researchers often adopt a hybrid architecture that integrates global and local planning. This hierarchical collaborative paradigm of “global guidance and local execution” has been recognized as the core framework for constructing intelligent autonomous systems and has been validated in numerous complex task planning domains. For instance, in the scenario of spacecraft autonomous avoidance of orbital debris, Chen et al. [20] developed an onboard autonomous system, the core of which is to guide the lower-level sensor scheduling and orbital maneuvering (micro-execution) through the upper-level task sequence planning (macro-decision), thereby achieving fully closed-loop autonomous operation. This work demonstrates that the deep integration of macroscopic strategic planning and microscopic real-time response is the key to enhancing the system’s adaptability in dynamically uncertain environments. Mapping this paradigm to the field of mobile robot navigation, the core challenge translates into how to achieve deep collaboration between global guidance at the geometric path level and local response at the motion control level. Against this backdrop, scholars have conducted extensive research to explore effective collaborative fusion mechanisms. For example, Li et al. [21] developed an integrated method by fusing their enhanced Genetic Algorithm with the DWA for route planning; through optimizing population diversity and the evaluation function, this method significantly enhances path smoothness and the robustness of dynamic obstacle avoidance, offering practical technical support for improving operational efficiency and ensuring operational safety in intelligent warehousing. However, the “brake and wait” strategy relied on by this fusion mechanism is essentially a passive response mechanism. It merely avoids collisions by stopping, lacking intelligent prediction of obstacle trajectories and failing to achieve efficient active bypass planning, which can easily lead to a decrease in operational efficiency or even cause system deadlock in dynamic dense or multi-robot collaborative scenarios. Yang et al. [22] proposed a dynamic trajectory planning method integrating the refined ant colony algorithm with the DWA. Through designing a deadlock handling strategy and refining the evaluation function, the approach remarkably boosts path search efficiency and motion stability, offering a practical approach to achieving autonomous navigation for mobile robots in cluttered, dynamic settings. However, its fusion strategy does not adaptively adjust according to the environmental spatial characteristics. In narrow and dense areas where the navigable space is severely compressed, the algorithm still maintains fixed evaluation weights for heading angle, speed, and safety distance. This results in insufficient safety redundancy and a lack of adaptability and robustness in non-uniform dynamic environments. Kim et al. [23] introduced a hierarchical strategy that employs the A* algorithm for global route planning and refines local trajectories through the Analytic Hierarchy Process (AHP), achieving optimal path generation for patient transport robots in hospital scenarios. However, its fusion strategy directly takes the original path generated by the traditional A* algorithm as global guidance without post-processing optimizations such as smoothing and pruning. The path itself has abrupt turns and closely follows obstacles, making the local AHP make decisions based on a suboptimal and unsafe global benchmark. As a result, the overall performance and riding comfort of the fusion algorithm are severely constrained. Luo et al. [24] proposed the ESSA-DWA dynamic path planning algorithm, which fuses the enhanced sparrow search algorithm (ESSA) with the DWA. By introducing Tent chaotic initialization and dynamically adjusting inertia weights, the algorithm significantly improves path search efficiency and adaptability to complex environments, enabling efficient autonomous navigation and real-time obstacle avoidance for mobile robots. However, the proposed ESSA-DWA fusion strategy only optimizes ESSA, while directly using the classic DWA algorithm as the local planner without any improvement to its evaluation function, obstacle avoidance strategy, etc. This results in the inherent problems of DWA, such as local optimum traps, obstacle avoidance oscillations, and insufficient dynamic adaptability, being fully retained, limiting the robustness and reliability of the overall fusion framework. Overall, existing hybrid methods tend to focus on simple integration at the algorithm level; there is still room for further improvement in terms of optimizing the tracking friendliness of global paths, deep coupling of local planning and global information, and other aspects.

Building on the limitations discussed earlier, we propose an Enhanced A*-DWA Fusion Algorithm (EA-DFA) to address the navigation challenge for mobile robots in dynamic, cluttered settings; the core innovations of this study are summarized as follows:

(1) Inspired by the phenomenon of biological path self-optimization, an innovative three-stage hierarchical optimization strategy is designed to address the core issues of traditional A* planning, such as excessive path inflection points, poor smoothness, and difficulty in tracking. Through the synergistic effect of redundant point reduction and bidirectional path relaxation, it achieves a balance between global optimality and local tracking friendliness, providing a high-quality reference benchmark for subsequent local planning.

(2) Drawing on the adaptive behavior mechanism of animals in dynamic obstacle avoidance, a two-dimensional enhancement scheme is proposed to address the core issues of traditional dynamic window methods, such as rigid initial heading angle setting, insufficient obstacle avoidance robustness, and poor environmental adaptability. This scheme involves dynamically calibrating the initial heading angle based on intermediate transition points along the global path, constructing an evaluation function that integrates dynamic obstacle perception, a safety distance saturation mechanism, and environmental adaptive weights, significantly enhancing the intelligence and safety of local obstacle avoidance.

(3) By mimicking the hierarchical decision-making logic of “strategy-execution” in biology, a collaborative fusion framework combining three-stage optimized A* and two-dimensional enhanced DWA is constructed. Through global optimal path guidance and real-time local target iteration mechanism, the enhanced local planner is driven to adaptively adjust the trajectory within the rolling window, achieving deep collaboration between global path-tracking friendliness and local obstacle avoidance dynamic responsiveness.

(4) Through dual verification of multi-scenario simulation experiments and physical experiments based on the ROS platform, the superiority of the proposed EA-DFA algorithm in dynamic complex environments has been systematically confirmed, providing a solution that combines theoretical innovation and engineering practicality for efficient and safe navigation of mobile robots.

To further clarify the research progress of the fusion algorithm combining A* and DWA, and to identify the similarities, differences, and advancements between the work presented in this paper and recent representative studies, Table 1 provides a systematic comparison between the EA-DFA proposed in this paper and three typical fusion algorithms presented in references [25,26,27], across multiple dimensions.

As shown in Table 1, existing research has contributed to enhancing path smoothness, safety, or introducing adaptive strategies, providing important references and guidance for this study. On this basis, the proposed EA-DFA in this paper further optimizes the path quality and tracking friendliness of global planning, strengthens the dynamic response capability of local planning, and simultaneously enhances the adaptability to environmental complexity for dynamic obstacle avoidance. Verified by simulation and physical experiments, this method can balance path optimality, motion smoothness, and environmental adaptability in complex dynamic environments, demonstrating engineering practicality.

The remainder of this paper is structured in the following manner: Section 2 elaborates on the path optimization strategy for improving the A* algorithm; Section 3 introduces the key technologies for improving the DWA algorithm; Section 4 designs the overall architecture and process of the fusion algorithm; Section 5 verifies the algorithm performance through multiple sets of simulation experiments; Section 6 verifies the algorithm performance through laboratory physical experiments; and Section 7 synthesizes the contributions of this work and proposes extensions for future investigation.

## 2. Enhanced A* Algorithm for Global Path Planning

### 2.1. Traditional A* Algorithm

The A* algorithm is renowned as a classic heuristic search method well suited for resolving path planning problems within globally known environments [28]. Its core mechanism lies in constructing a heuristic cost function by utilizing known environmental map information, thereby guiding the search process to efficiently advance towards the target direction. Compared to blind search algorithms such as Dijkstra [29] and Floyd [30], this algorithm significantly reduces the number of nodes that need to be expanded by introducing heuristic estimation, thus greatly improving search efficiency while approaching optimal paths if certain prerequisites are met. The cost function is expressed as [9]:

(1) f(m)=g(m)+h(m)

In which, m denotes the current node; g(m) denotes the actual cumulative cost of the mobile robot moving from the Start to m; and h(m) denotes the estimated cost from m to the Target. As a key part of the function, its proper setting is crucial for achieving rapid convergence and a near-optimal path [31].

Common calculation methods for the estimated cost h(m) include Manhattan distance, Euclidean distance, and Chebyshev distance. This study conducts relevant research on complex map environments containing static and dynamic obstacles. In such environments, the mobile robot needs to achieve efficient collision-free navigation through a combination of diagonal and horizontal movements. Due to the motion trajectory being constrained by obstacles and influenced by multi-directional movement, the actual path length is usually longer than the shortest straight-line distance characterized by Euclidean distance but less than the strictly orthogonal path length characterized by Manhattan distance. Therefore, this study employs the following formula for calculating the estimated cost [32]:

(2) $$h(m)=\sqrt{2}\times \min\{d_{y},d_{x}\}+(d_{y}+d_{x}-2\times \min\{d_{y},d_{x}\})$$

where $d_{y}=\left|y_{m}-y_{T\arg et}\right|$ represents the vertical distance from the current point m to the Target point; $d_{x}=\left|x_{m}-x_{T\arg et}\right|$ represents the horizontal distance from the current point m to the Target point.

### 2.2. Three-Stage Optimization Strategy

The A* algorithm is renowned as a classic heuristic search method, well suited for resolving path planning problems within globally known environments. Its core mechanism lies in constructing a heuristic cost.

The path planned by the traditional A* algorithm in a grid map is formed by connecting the centers of adjacent grids, exhibiting a piecewise linear characteristic [33]. The path (defined as the original path) suffers from issues such as excessive redundant nodes, frequent turns, and insufficient smoothness, which are not conducive to the mobile robot achieving efficient and stable movement. In nature, organisms such as honeybees and desert ants have evolved efficient path optimization strategies over long-term evolution; they actively eliminate redundant segments in their foraging and homing routes and smooth trajectory inflection points to complete navigation tasks with minimal energy consumption, and their core logic lies in balancing path conciseness and motion efficiency [34]. Inspired by this biological navigation intelligence, a three-stage optimization strategy is proposed in this paper to address the aforementioned shortcomings of the traditional A* path, with the specific steps outlined as follows:

#### 2.2.1. Once-Optimization: Redundant Point Deletion

Assume that the original path planned by the traditional A* algorithm from the Start to the Target is $\left[P_{1},P_{2},P_{3},...,P_{n}\right]$. Starting from the initial point, we sequentially judge whether points $P_{1}$, $P_{2}$, and $P_{3}$ are collinear along the path: if they are collinear, $P_{2}$ is identified as a redundant point to be deleted, and then points $P_{1}$, $P_{3}$, and $P_{4}$ are examined; if they are not collinear, construct a rectangular area with $P_{1}$ and $P_{4}$ as the diagonal vertices, and judge whether the shortest distance h from obstacles within this rectangular area to the straight line 1→4 is greater than the Safe_Distance; if it is, point 3 is also identified as a redundant point, and then points $P_{1}$, $P_{4}$, and $P_{5}$ are examined. The above judgment process is repeated until all points in the path are traversed; the path after the first optimization is referred to as the Once-Optimized Path. The pseudocode is as follows (Table 2):

#### 2.2.2. Twice-Optimization: Forward Path Relaxation

Although Once-Optimization has eliminated redundant points from the original path, achieving path simplification by retaining only necessary key turning points, the following issues still persist: Firstly, the path length is not geometrically optimal. The simplified path is merely the shortest path under the constraint of “sequentially passing through the remaining key turning points,” and its line segment connection does not fully utilize map environmental information, nor is it the globally shortest path from the Start to the Target. Secondly, the motion smoothness is insufficient. The sharp corners formed at key turning points of the path require the mobile robot to perform intermittent movements of “stopping-turning-then accelerating” at these points. This not only reduces movement efficiency and increases energy consumption but also poses challenges to the control accuracy and response performance of the underlying motion control system.

To address the aforementioned issues, this study further introduces a path relaxation strategy based on redundant point elimination. The core idea is as follows: Let Once-Optimized Path = $\left[P_{1},P_{2},P_{3},...,P_{n}\right]$. Select three consecutive points $P_{i}$, $P_{i+1}$, and $P_{i+2}$ on the path in sequence as a processing unit, then attempt to bypass the intermediate turning point $P_{i+1}$, and search forward for the optimal intermediate transition point on the line connecting $P_{i}$ and $P_{i+2}$, thereby achieving path shortening and corner smoothing (eliminating sharp corners). Specifically, the algorithm first samples the line connecting points $P_{i}$ and $P_{i+2}$ at a fixed step size σ to generate a series of candidate points, Candidates = $\left[C_{1},C_{2},C_{3},...,C_{k}\right]$, and starts traversing in the direction from $P_{i}$ to $P_{i+2}$. For each candidate point, the algorithm performs collision detection to determine whether its distance to the nearest obstacle satisfies the minimum safety distance constraint. Once a qualified candidate point $C_{j}$ is found, it is immediately selected as the optimal intermediate transition point to replace $P_{i+1}$ in the original path, thereby optimizing the original path segment $P_{i}$→$P_{i+1}$→$P_{i+2}$ into a shorter and smoother segment $P_{i}$→$C_{j}$→$P_{i+2}$. After completing the optimization of the current unit, the algorithm slides to the next triplet window based on the updated path points and repeats the above path relaxation operation until the entire path is traversed. The pseudocode is as follows (Table 3):

We define the path after the second optimization as Twice-Optimized Path. The core objective of this optimization is to select the optimal intermediate transition point through a forward path relaxation strategy. On the one hand, it generates a new path with gentler turning angles. Although the path is still composed of polylines, its smoothness is significantly improved, which can effectively reduce the difficulty of motion control for the mobile robot and decrease energy consumption. On the other hand, it further shortens the total path length, making it closer to the globally shortest path.

#### 2.2.3. Thrice-Optimization: Reverse Path Relaxation

Based on the Twice-Optimized Path obtained via the forward path relaxation described earlier, this study further proposes a reverse optimization strategy to eliminate local redundancies that may be left by a single forward traversal, thereby comprehensively enhancing the smoothness and overall quality of the path. The core idea of this strategy is consistent with forward optimization, but the execution direction is reversed. That is, the algorithm starts from the end of the path and traverses the point sequence in reverse to search for the optimal intermediate transition point. The reverse optimization process complements the forward process, enabling the path to be “straightened” from different directions, further shortening the total path length and increasing the turning angles. After the third optimization (defined as the Thrice-Optimized Path), the length of the new path is further reduced, and the turning angles at certain turning point is further increased.

### 2.3. Workflow of the Enhanced A* Algorithm

**Figure 1:**
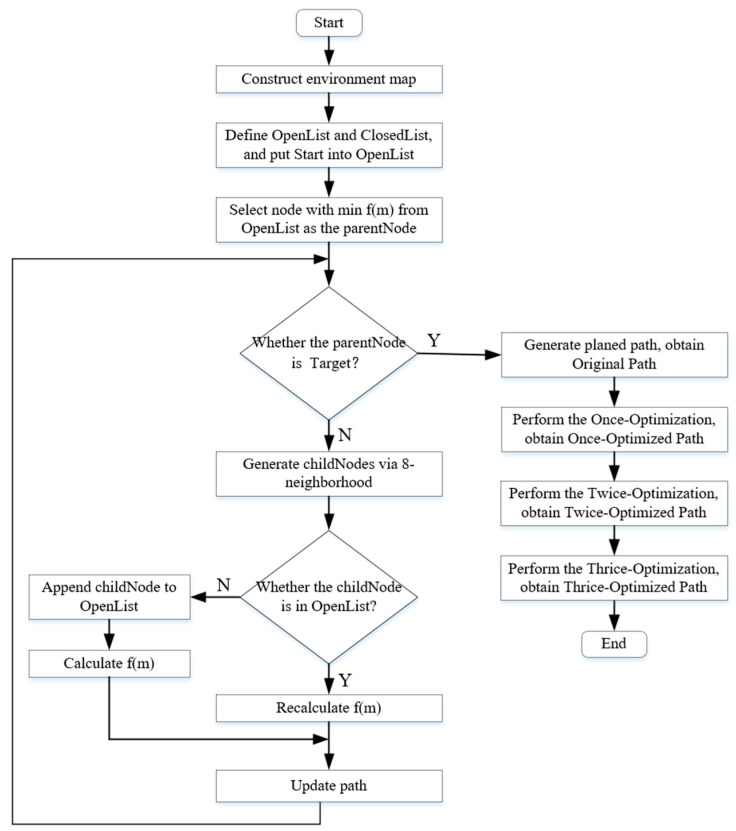
Execution flowchart of the enhanced A* algorithm.

The complete execution process of the enhanced A* algorithm proposed in this study encompasses the path search of the traditional A* algorithm and the subsequent three-stage optimization strategies, as illustrated in Figure 1. The specific steps are as follows:

Step 1: Construct the environmental map and set the Start point and Target point.

Step 2: Define two lists: OpenList to store nodes to be explored, and CloseList to store nodes that have been explored, and place Start into OpenList.

Step 3: Select the node with the minimum cost value f(m) from OpenList as the current parentNode, add it to CloseList, and remove it from OpenList.

Step 4: Determine whether the parentNode is the Target. If it is, terminate the search and generate the planned path; otherwise, generate its childNodes using the 8-neighborhood method.

Step 5: Check whether each childNode is in the OpenList. If it is, recalculate the cost value f(m), compare it with the former, retain the smaller value, and update the path; if not, append it to the OpenList, calculate its cost value f(m), and update the path.

Step 6: Return to Step 3 to perform the cyclic search until the Target is found, obtaining the original path.

Step 7: Perform the Once-Optimization to remove all redundant nodes in the path, retaining only the necessary key turning points, resulting in the Once-Optimized Path.

Step 8: Perform the Twice-Optimization, smooth the path using the forward relaxation strategy, resulting in the Twice-Optimized Path.

Step 9: Perform the Thrice-Optimization, further smooth the path using the reverse relaxation strategy, resulting in the Thrice-Optimized Path.

## 3. Improved DWA for Local Path Planning

### 3.1. Traditional DWA

The DWA is a local path planning algorithm based on velocity space sampling [35]. Its core mechanism lies in generating a large number of candidate velocity pairs (v,ω) within the velocity space satisfying multiple constraints in each sampling period Δt, and these candidate velocity pairs collectively constitute a dynamic window. The construction of this window requires sequentially considering two types of constraints, and then through steps including trajectory deduction, multi-objective evaluation, and optimal selection, the robot’s motion state at the next moment is ultimately determined.

• Constraint Construction

The constraint construction of the dynamic window follows a logical progression from physical feasibility to environmental safety and sequentially encompasses the following two types of constraints:

Firstly, from the perspective of physical feasibility, based on the kinematic limits (maximum/minimum linear velocity) and dynamic constraints (maximum acceleration/maximum deceleration) of the robot, the range of admissible linear and angular velocities is defined:

Admissible Linear Velocity Set:

$$V_{admissible}=\left\{\left.v\right|a\leq v\leq b\right\}$$

(3) $$a=\max(v_{\min},v_{t}-\overset{\cdot }{v_{a}}*\Delta t)$$

$$b=\min(v_{\max},v_{t}+\overset{\cdot }{v_{a}}*\Delta t)$$

where $v_{\min}$ and $v_{\max}$ represent the lower and upper bounds of v, respectively; $v_{t}$ denotes the current linear velocity of the robot; and $\overset{\cdot }{v_{a}}$ signifies the maximum linear acceleration/deceleration of the robot.

Admissible Angular Velocity Set:

$$\omega_{admissible}=\left\{\left.\omega \right|c\leq \omega \leq d\right\}$$

(4) $$c=\max(\omega_{\min},\omega_{t}-\overset{\cdot }{\omega_{a}}*\Delta t)$$

$$d=\min(\omega_{\max},v_{t}+\overset{\cdot }{\omega_{a}}*\Delta t)$$

where $\omega_{\min}$ and $\omega_{\max}$ represent the lower and upper bounds of ω, respectively; $\omega_{t}$ denotes the current angular velocity of the robot; and $\overset{\cdot }{\omega_{a}}$ signifies the maximum angular acceleration/deceleration of the robot.

Secondly, to ensure safety, environmental safety constraints need to be introduced. The velocity pairs (v,ω) filtered above only ensure the physical feasibility of linear and angular velocities, and do not account for environmental safety. This constraint ensures the robot has reliable emergency braking capability at any point on the predicted trajectory by requiring its braking distance to be less than the minimum distance to obstacles (denoted as dist(v,ω)). The mathematical expression is as follows:

(5) $$v\leq \sqrt{2\cdot \overset{\cdot }{v_{a}}\cdot dist(v,\omega )}$$

• Trajectory Deduction

**Figure 2:**
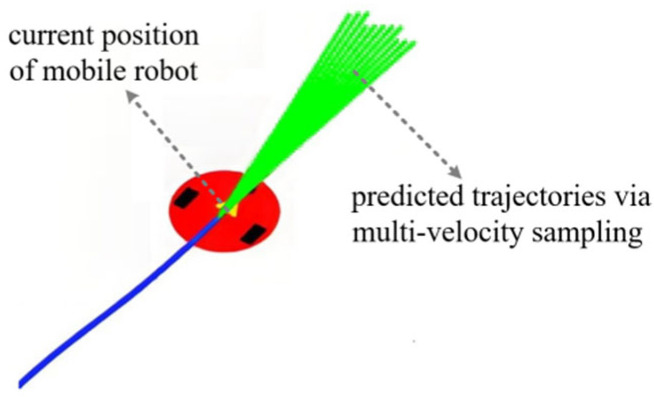
Predicted trajectories within the dynamic window.

For each retained candidate velocity pair (v,ω), trajectory deduction is performed based on the robot’s kinematic model (Equation (6)) to predict the motion trajectory within a future time period $T_{sim}$, as shown in Figure 2.

(6) $$\left\{\begin{array}{l} x_{t+1}=x_{t}+v\cdot \Delta t\cdot \cos(\theta_{t})\\ y_{t+1}=y_{t}+v\cdot \Delta t\cdot \sin(\theta_{t})\\ \theta_{t+1}=\theta_{t}+\omega \cdot \Delta t \end{array}\right.$$

where $\left(x_{t},y_{t}\right)$ and $\left(x_{t+1},y_{t+1}\right)$ represent the positions of the robot at time t and t+1, respectively; $\theta_{t}$ and $\theta_{t+1}$ denote the yaw angles of the robot at time t and t+1, respectively; and Δt is the sampling period.

For each candidate velocity pair (v,ω), the complete trajectory deduction calculation follows the following steps:

(1) Initialization: Take the robot’s current global pose $P_{0}=(x_{0},y_{0},z_{0})$ as the starting point for the simulation. Set the sampling period Δt and the total simulation time $T_{sim}$, so the total number of simulation steps is $N=T_{sim}/\Delta t$. Initialize the trajectory sequence $P=\left\{P_{0}\right\}$.

(2) Iterative prediction: Perform a loop on t=0,1,2,...,N−1:

① Let the current pose be denoted as $P_{t}=(x_{t},y_{t},z_{t})$.

② Calculate the pose $P_{t+1}=(x_{t+1},y_{t+1},z_{t+1})$ at the next moment based on the kinematic model (6).

③ Add the new pose to the trajectory sequence: $P\leftarrow P\cup \left\{P_{t+1}\right\}$.

(3) Output trajectory: After the loop ends, the predicted trajectory $P=\left\{P_{0},P_{1},P_{2},...,P_{N}\right\}$ is obtained, which consists of N+1 number of discrete pose points and represents the robot’s motion path within $T_{sim}$ future time interval under the control of speed (v,ω).

• Multi-Objective Evaluation

After completing the trajectory deduction for all candidate velocity pairs, a multi-objective evaluation function (Equation (7)) is used to quantitatively assess these predicted trajectories, in order to select the optimal speed pair.

(7) $$G(v,\omega )=\alpha \cdot head_{norm}(v,\omega )+\beta \cdot dist_{norm}(v,\omega )+\gamma \cdot vel_{norm}(v,\omega )$$

where α, β, and γ are weight coefficients used to adjust the relative importance of various indicators. $head_{norm}(v,\omega )$, $dist_{norm}(v,\omega )$, and $vel_{norm}(v,\omega )$ represent the normalized values of head(v,ω), dist(v,ω), and vel(v,ω), respectively.

The definitions of each evaluation sub-function are as follows:

Guidance sub-function head(v,ω): represents the alignment degree of the trajectory end with the target direction [36], with its value being as follows:

(8) $$head(v,\omega )=180{}^{\circ}-\left|\theta_{goal}-\theta_{traj}\right|$$

where $\theta_{goal}$ is the azimuth angle from the trajectory end to the Target point, and $\theta_{traj}$ is the yaw angle of the robot at the trajectory end.

Safety sub-function dist(v,ω): Takes the distance from the end of the current predicted trajectory to the nearest obstacle.

Velocity sub-function $vel_{norm}(v,\omega )$: Takes the current linear velocity v.

Finally, the velocity pair with the highest score in the evaluation function G(v,ω) is selected as the linear and angular velocities of the mobile robot for the next moment.

### 3.2. Initial Heading Angle Setting

**Figure 3:**
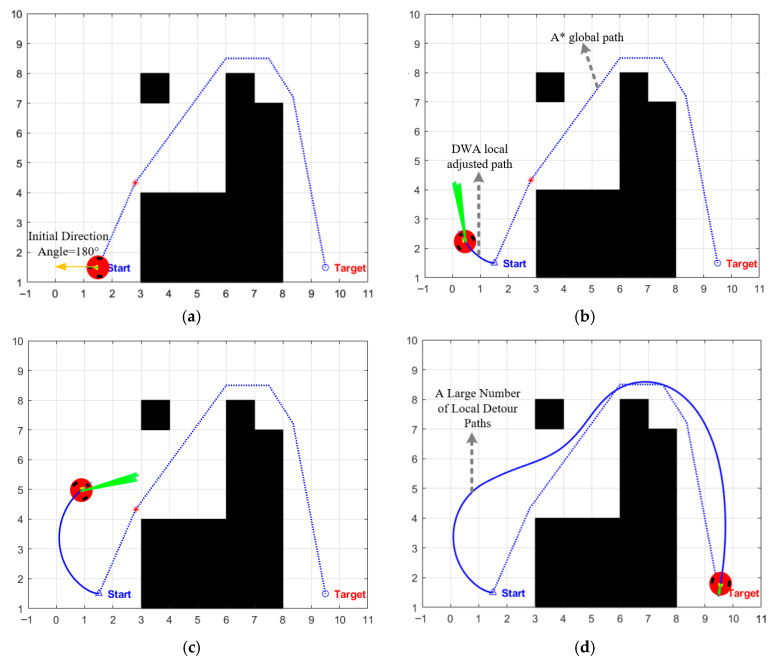
Path planning with fixed initial heading angle: (**a**–**d**) Snapshots illustrating different stages of the path planning process.

At the beginning of the first iteration of the DWA, an initial heading angle must be set for the robot, which directly determines the starting direction of the first batch of simulated trajectories. In traditional DWA for path planning, the initial heading angle is usually assigned a fixed value (such as 0°, +90°, or 180°). However, this setting has obvious drawbacks: when the Target point is located on the side or behind the robot’s initial orientation, the robot often needs to waste time rotating in place or taking local detours to gradually adjust to the target direction, thereby significantly reducing the execution efficiency of path planning. As shown in Figure 3, due to the large deviation between the initial heading angle and the target direction, the robot’s planned trajectory exhibits significant local detours in the initial stage, requiring additional path adjustments to correct the direction of travel. This intuitively reflects the decrease in execution efficiency under this strategy.

**Figure 4:**
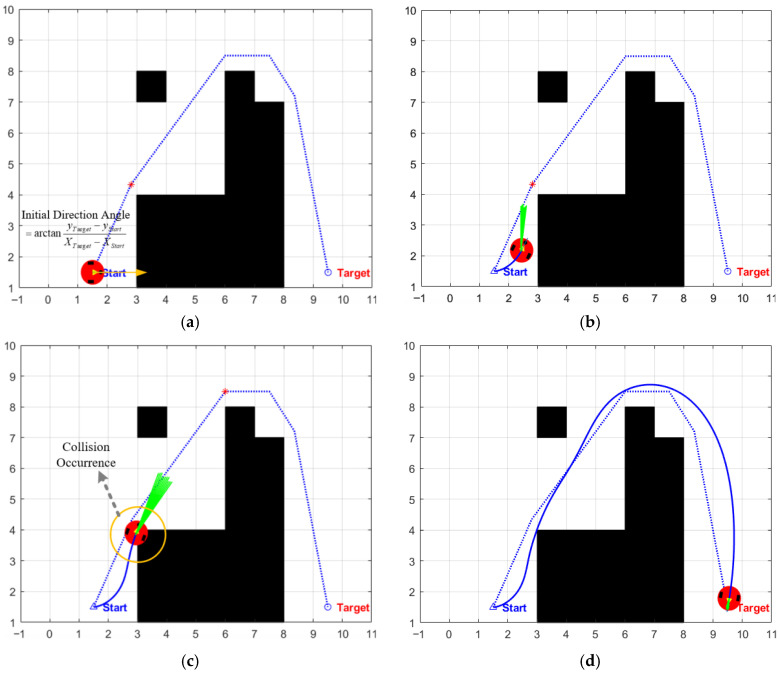
Path planning with initial heading angle from Start to Target: (**a**–**d**) Snapshots illustrating different stages of the path planning process.

To address this issue, some studies have proposed an improved scheme, which sets the initial heading angle by calculating the azimuth between the starting point and the global target point [37]. Although this method incorporates global target information, it ignores the real-time constraints of the local environment, which may lead to serious consequences in complex scenarios. For instance, if an obstacle lies on the line connecting the starting point and the global target point and is adjacent to the starting point, the preset heading angle will guide the evaluation function to prioritize trajectories along this line, which directly points toward the obstacle. This not only poses a collision risk but may also lead to the planning process falling into local deadlock and failing. As shown in Figure 4, there is an obstacle in the direction of the global target point, the trajectory approaches the obstacle area at the initial stage and incurs a significant collision with the obstacle during the planning process.

**Figure 5:**
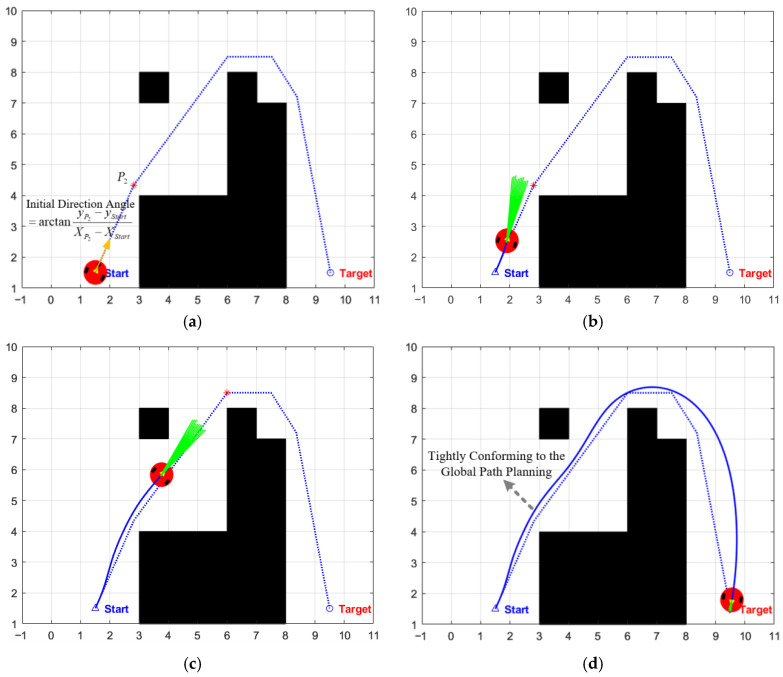
Path planning with initial heading angle from Start to $P_{1}$: (**a**–**d**) Snapshots illustrating different stages of the path planning process.

To tackle the deficiencies of the aforementioned two methods, this paper proposes an initial heading angle optimization strategy based on global path guidance. Firstly, the enhanced A* algorithm proposed in this paper is utilized for global path planning in a known static map, generating an optimal path composed of a series of key intermediate transition points, which is $\left[Start,P_{1},P_{2},P_{3},...,P_{n},Target\right]$. Subsequently, in the initialization phase of DWA local planning, the first intermediate transition point $P_{1}$ of the global path is set as the initial local goal, and the azimuth angle between Start and $P_{1}$ is calculated. This angle is directly adopted as the initial heading angle for the first iteration of the DWA algorithm. This improvement ensures that from the first planning cycle onwards, the starting direction of the simulated trajectory points toward the first intermediate transition point of the global optimal path, thereby providing a correct and efficient initial search direction for the subsequent iterative planning of the dynamic window. As shown in Figure 5, the trajectory follows the extension trend of the global path from its starting position, thereby ensuring that the planning is both globally optimal and safe from the initial stage.

### 3.3. Evaluation Function Optimization

In local path planning for mobile robots based on the DWA, the safety sub-function dist(v,ω) plays a crucial role in ensuring collision-free navigation for the robot [38]. The traditional DWA algorithm has three limitations in the design of this sub-function: Firstly, when calculating the distance from the trajectory endpoint to the nearest obstacle, it only considers the impact of static obstacles and does not incorporate the distance evaluation of dynamic obstacles. Secondly, the sub-function always adopts the Euclidean distance from the trajectory endpoint to the obstacle as the evaluation score. In open environments, this distance value is likely to be excessively large, disrupting the balance between the guidance evaluation and velocity evaluation, leading to an overly conservative planning strategy and thus sacrificing navigation efficiency. Thirdly, its safety evaluation weight β is usually set as a fixed value and cannot be adaptively adjusted according to the congestion degree of the local environment [39], resulting in a lack of flexibility in the planning strategy.

In response to the aforementioned issues, this paper has made three targeted improvements to the safety sub-function:

• Integration of a Dynamic Obstacle Model

To enhance the algorithm’s obstacle avoidance capability in dynamic environments, this paper extends the obstacle model. In traditional methods, the obstacle set Obs_static only includes static obstacles. This study expands it to the union of static and dynamic obstacles, that is, Obs = Obs_static∪Obs_dynamic. When calculating the safety sub-function dist(v,ω) for the current predicted trajectory, the algorithm will search for the minimum value in the extended obstacle set. This improvement enables the robot to perceive and respond to moving obstacles, thereby enhancing the foresight and safety of the navigation process.

• Establishment of a Safety Distance Saturation Mechanism

To address the issue of evaluation function imbalance caused by an excessively large value of dist(v,ω) in open environments, this paper introduces a saturation mechanism: First, set a saturation threshold (here set to 2⋅Safe_Distance). When the calculated dist(v,ω) is greater than this threshold, the value of dist(v,ω) is limited to this saturation threshold. The core purpose of this mechanism is to prevent the safety evaluation term from occupying an absolutely dominant position in the overall evaluation function, thereby ensuring that the guidance evaluation term and the velocity evaluation term can play their due roles in the decision-making process and maintaining the balance of the algorithm in multi-objective optimization.

**Figure 6:**
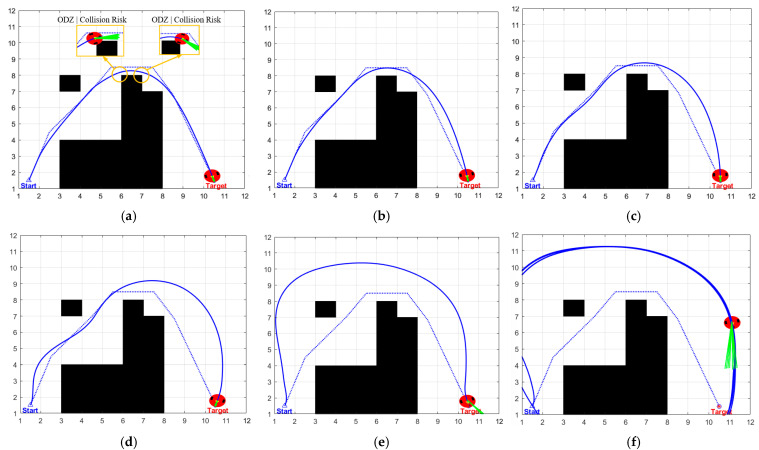
Path planning effects under different safety distance saturation thresholds: (**a**) corresponds to 0.5⋅Safe_Distance; (**b**) corresponds to 1⋅Safe_Distance; (**c**) corresponds to 2⋅Safe_Distance; (**d**) corresponds to 3⋅Safe_Distance; (**e**) corresponds to 4⋅Safe_Distance; (**f**) corresponds to 5⋅Safe_Distance.

To verify the rationality and optimality of the safety distance saturation threshold, this study designs a parameter sensitivity comparison experiment: in the same map scenario, other algorithm parameters are fixed, and only the safety distance saturation threshold is adjusted. A total of six sets of variables are set, which are 0.5 times, 1 time, 2 times, 3 times, 4 times, and 5 times the safety distance, respectively. Each group of experiments is run independently 30 times. The experimental results are presented in Table 4, and the corresponding path planning results are illustrated in Figure 6a–f.

Observations from Table 4 reveal that as the safety distance saturation threshold increases from 0.5 times to 4 times, the data in the table shows a gradual decrease in planning time from 37.4450 s to 18.2190 s, while the path length increases from 17.0221 m to 23.4629 m. Figure 6a–e visually demonstrate a continuous decrease in the fit between the planned path and the global optimal path. Specifically, the path at 0.5⋅Safe_Distance is the shortest, but as evident from Figure 6a, there are two collisions, posing serious safety risks and failing to meet application requirements; at the 1⋅Safe_Distance, no collisions occur, and the path fits well with the global optimal path (Figure 6b), but the planning time reaches 27.0140 s, indicating low efficiency; the planning times at the 3 and 4 times Safe_Distance are further shortened, but the path length significantly increases, and Figure 6d,e show obvious path redundancy and poor fit with the global optimal path; at the 5⋅Safe_Distance, due to a severe imbalance in the evaluation function, the robot rotates in place to maintain an excessively large safety distance, completely neglecting the core task of moving towards the target point. It did not generate a path to reach the target even after one minute. In contrast, the 2⋅Safe_Distance achieves optimal balance among multiple objectives: firstly, the planned path is safely reachable, and secondly, the planning time is 23.2600 s (a reduction of 13.9% compared to 1⋅Safe_Distance), and the path length is only 18.1070 m (an increase in only 3.3% compared to 1⋅Safe_Distance). This unique advantage of simultaneously considering safety, efficiency, and path optimality fully validates the scientific and rational setting of the 2⋅Safe_Distance saturation threshold.

• Design of an Adaptive Strategy for Safety Evaluation Weight

To enable the planning strategy to intelligently respond to environmental changes, this paper designs a weight adjustment strategy based on local environmental features. This design is inspired by the adaptive intelligence of natural animals such as antelopes and bats in dynamic obstacle avoidance—these creatures adjust their behavioral weights in real time based on local environmental features such as obstacle density and target distance when navigating complex environments. In open, low-threat scenarios with few obstacles, they prioritize increasing the weight of movement efficiency to quickly complete foraging or migration tasks. In scenarios with dense obstacles and imminent danger, they immediately emphasize the weight of obstacle avoidance safety, reduce movement speed, and precisely evade risks, ultimately achieving a dynamic balance between efficiency and safety [40]. Inspired by this, this paper integrates this biological adaptive mechanism into the weight design of the dynamic window method. This strategy dynamically adjusts the weight coefficient β of the safety evaluation term by calculating the obstacle occupancy rate ρ of the 8-neighborhood grids around the endpoint of the current predicted trajectory. When ρ>0.25, it indicates that the robot is in a relatively complex area; at this time, the weight is increased according to (Equation (9)) to enhance obstacle avoidance:

(9) $$\beta =\beta_{0}\cdot 2\cdot (1+\rho )$$

Otherwise, the base weight $\beta_{0}$ is adopted to encourage efficient travel of the robot. This design enables the algorithm to autonomously adjust its behavioral strategy according to the congestion degree of the environment. In congested environments, the weight of the safety term is proactively increased, prompting the robot to adopt a more conservative obstacle avoidance strategy to ensure navigation safety; in open environments, the base weight provides the robot with greater motion freedom, achieving the synergistic improvement of target orientation and motion efficiency.

To verify the effectiveness of the proposed adaptive weight strategy, this paper designs four sets of comparative experiments in the same narrow corridor environment with multiple obstacles. The experiments maintain the core parameters, such as the fixed basic weight $\beta_{0}$ and the obstacle occupancy calculation rule, unchanged. By setting differentiated safety weight adjustment strategies, the performance differences between the fixed weight and adaptive weights with different adjustment magnitudes are compared. The safety weight calculation rules for the four sets of experiments are as follows:

Fixed weight strategy: $\beta =\beta_{0}$ (no adaptive adjustment is performed throughout the process, maintaining the base weight.)

Adaptive adjustment strategy 1: $\beta =\beta_{0}\cdot (1+\rho )$

Adaptive adjustment strategy 2 (adopted in this paper): $\beta =\beta_{0}\cdot 2\cdot (1+\rho )$

Adaptive adjustment strategy 3: $\beta =\beta_{0}\cdot 3\cdot (1+\rho )$

**Figure 7:**
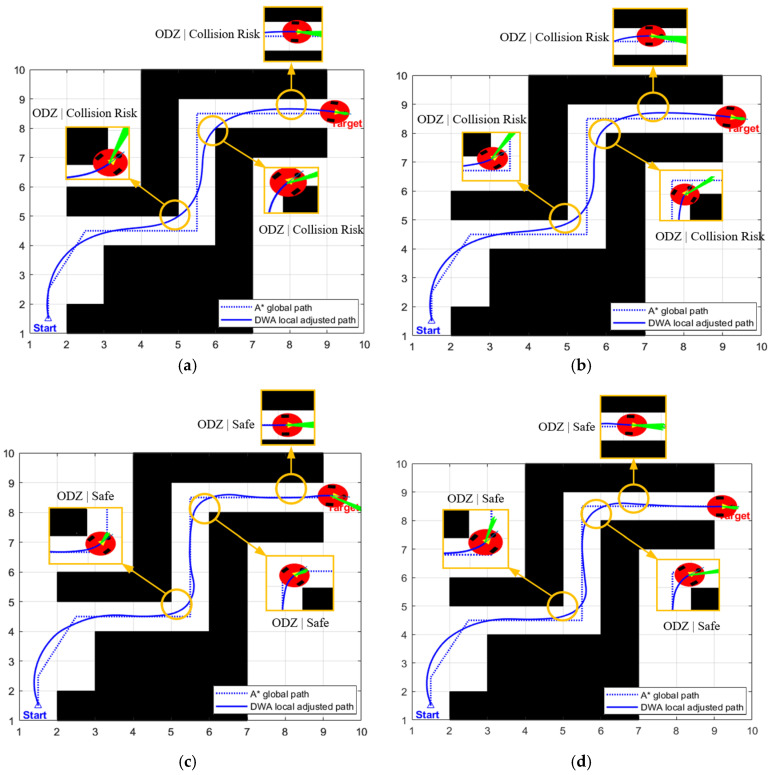
Path planning results under different safety weight adjustment strategies: (**a**) corresponds to the fixed weight strategy; (**b**) corresponds to adaptive adjustment strategy 1; (**c**) corresponds to adaptive adjustment strategy 2; (**d**) corresponds to adaptive adjustment strategy 3.

**Figure 8:**
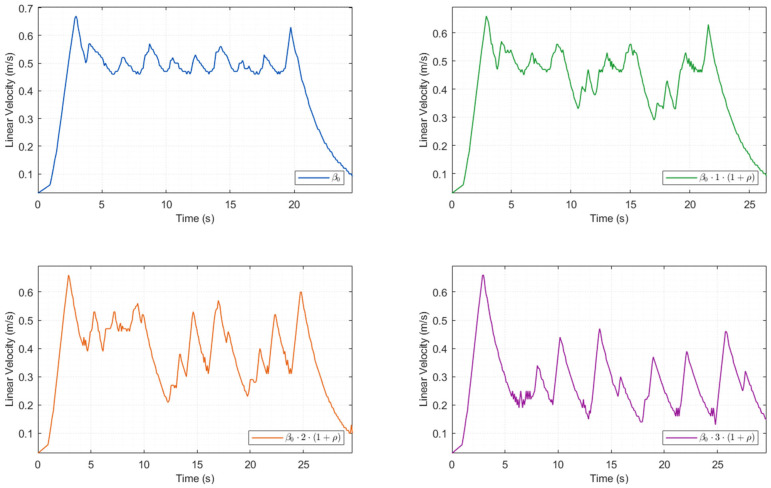
Time-series variations in robot linear velocity under different safety weight adjustment strategies.

Each set of experiments was independently run 30 times. The quantitative experimental results are presented in Table 5, the corresponding path planning results are illustrated in Figure 7, and the time-series variations in the robot’s linear velocity are depicted in Figure 8.

From the perspective of navigation safety, the fixed weight strategy ($\beta =\beta_{0}$) resulted in three significant collisions in the high-risk areas of ODZ (ODZ, Obstacle Danger Zone—refers to key areas with high collision risks for robots to pass through, uniformly marked with orange boxes in Figure 7a–d). Its extremely high collision risk renders it completely impractical for real-world applications. Although the adaptive strategy 1 ($\beta =\beta_{0}\cdot (1+\rho )$) was adjusted based on the obstacle occupancy rate, the adjustment magnitude was insufficient, and collisions still occurred in the aforementioned high-risk areas (as shown Figure 7b), thus failing to deliver adequate safety assurance. However, both strategy 2 and strategy 3 achieved zero collisions, successfully avoiding the collision risks in all high-risk areas of ODZ (see Figure 7c,d).

From the perspective of planning efficiency, as shown in Figure 8, the fixed weight strategy maintains a constant safety weight throughout the process without introducing a dynamic weight adjustment mechanism, and makes no targeted adjustments even when passing through the areas of ODZ. Therefore, it traverses the narrow corridor at a relatively high speed, achieving the highest average speed of 0.4435 m/s and the shortest planning time of 23.5620 s. However, the high speed directly leads to a sharp increase in collision risk. Strategy 1, owing to a slight increase in safety weight in accordance with the obstacle occupancy rate, travels through the narrow corridor at a marginally lower speed than the fixed weight strategy, with the average speed dropping to 0.4209 m/s and the planning time increasing to 25.5870 s. Although the speed is reduced, the collision problem is not solved. Adaptive adjustment strategy 3, due to excessive adjustment of safety weight, causes the algorithm to overly focus on obstacle avoidance safety in path planning, maintaining a very low speed throughout the process, with an average speed of only 0.2745 m/s and a planning time of up to 39.8800 s. Although safety is guaranteed, efficiency is excessively sacrificed, severely restricting the robot’s operational efficiency. Adaptive adjustment strategy 2 tends to be conservative when passing through the narrow corridor, with an average speed of 0.3825 m/s and a planning time of 29.6180 s. Compared to the fixed strategy, the path length increases by 5.6% and the planning time increases by 25.7%, with the efficiency loss being within a controllable range. Compared to strategy 3, the average speed increases by 39.3% and the planning time decreases by 25.7%. While achieving zero collisions, it significantly improves operational efficiency, truly achieving a balanced trade-off between safety and efficiency. In summary, this paper selects the adaptive adjustment strategy 2.

## 4. Fusion of the Enhanced A* and DWA

**Figure 9:**
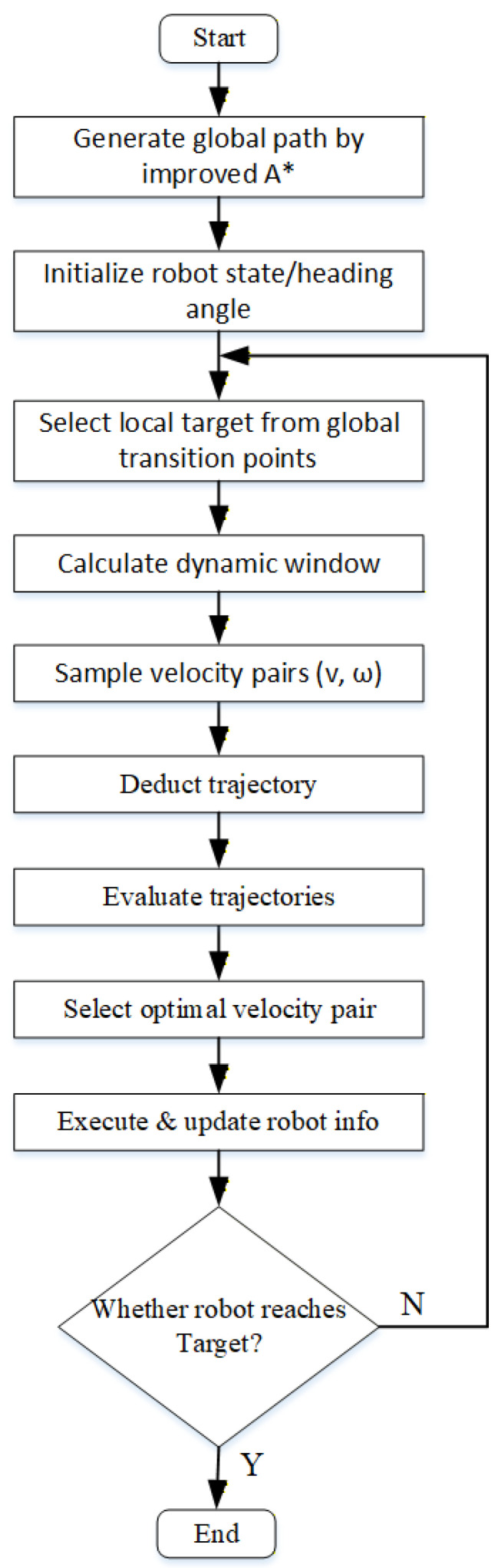
Execution flowchart of the proposed EA-DFA.

To overcome the limitations of single planning algorithm and achieve the unification of global path optimality and local real-time obstacle avoidance, we deeply integrate the enhanced A* algorithm with the improved DWA to construct a collaborative navigation framework. This framework uses the optimized global path generated by the former as a guide and the latter as a local rolling decision-making and execution unit. By dynamically taking the intermediate transition points on the global path as local goals in real time, the DWA is driven to perform trajectory optimization and dynamic obstacle avoidance within the rolling window. This ensures that the local motion of the robot always remains consistent with the global direction, while being able to flexibly respond to dynamic environmental changes, thereby improving the overall smoothness, safety, and efficiency of navigation. The specific integration process is illustrated in Figure 9 as follows:

Step 1: Global Path Generation

Generate the global path based on the enhanced A* algorithm and obtain key intermediate transition points.

Step 2: Robot Initialization

Set the initial state, initial heading angle, and other relevant information of the robot.

Step 3: Local Target Point Selection

From the sequence of global intermediate transition points, select the node that is closest to the robot’s current position and located in front of it as the current local target point.

Step 4: Dynamic Window Calculation

Based on the robot’s current velocity, acceleration, and safety constraints, calculate the range of actually reachable linear and angular velocities for the next time interval.

Step 5: Velocity Sampling

Within the dynamic window range, perform discrete sampling on the linear velocity v and angular velocity ω to generate a series of candidate velocity pairs (v,ω).

Step 6: Trajectory Deduction

For each sampled velocity pair (v,ω), deduct the motion trajectory for a period of $T_{sim}$ into the future based on the robot’s kinematic model.

Step 7: Trajectory Evaluation

Score each simulated trajectory according to Equation 9 and combine with the optimization of the evaluation function described in Section 3.3.

Step 8: Optimal Velocity Pair Selection

From all candidate trajectories, select the velocity pair corresponding to the trajectory with the highest evaluation function score as the optimal control command at the current moment.

Step 9: Execution and Update

Send the optimal velocity command to the robot’s underlying controller to drive the robot to move for one control cycle and update its pose and environmental information.

Step 10: Loop Judgment

Check whether the robot has reached the Target (The straight-line distance between the current location and the Target is less than 0.2 m). If not, return to Step 3 to continue executing local rolling planning; if yes, the planning ends.

## 5. Algorithm Validation in Simulation

### 5.1. Parameter Settings

The EA-DFA algorithm proposed in this paper mainly comprises three categories of parameters: the basic parameters of the DWA algorithm, the mechanical performance parameters of the mobile robot, and the weight coefficients of the evaluation function. Among these, the mechanical performance parameters (e.g., maximum linear velocity, angular acceleration) are strictly configured in accordance with the technical specifications of the actual ROS-based mobile robot platform adopted by our laboratory, so as to ensure perfect consistency between the simulation conditions and the physical experimental setup. For the basic parameters of the DWA algorithm, the safety distance (0.75 m) is determined by combining the experimental environment (1 m grid cell size) and the robot’s physical dimensions (0.8 m diameter) to ensure sufficient collision avoidance margin; the sampling period (0.1 s) and total simulation time (3.0 s) are set with reference to the parameter ranges of mainstream application literatures [41,42] in relevant fields, and this parameter combination has been verified to achieve good planning performance through tests. Regarding the weight coefficients of the evaluation function (α = 0.05, $\beta_{0}$ = 0.1, γ = 0.2), we first referred to the parameter ranges set in existing studies on A*-DWA fusion algorithms [41,42,43], then fine-tuned them in conjunction with the characteristics of the EA-DFA algorithm proposed in this paper, ultimately selecting the parameter set with the best comprehensive performance. All parameter details are summarized in Table 6.

### 5.2. Experimental Simulation Environment

**Figure 10:**
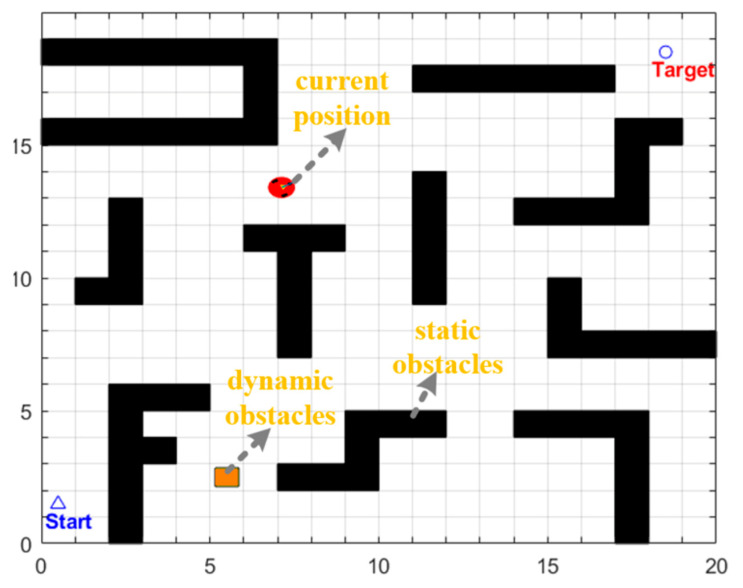
Map environment.

This experiment is based on the MATLAB R2022b platform for simulation verification. The experimental scene is built using a grid map [44], as shown in Figure 10. The side length of each grid cell was uniformly set to 1 m. Among them, black grids represent static obstacles, and white areas are defined as passable regions. The starting and ending points are marked with *Start* and *Target* for position calibration, respectively. Orange squares with a side length of 0.8 m were used to simulate dynamic obstacles.

**Figure 11:**
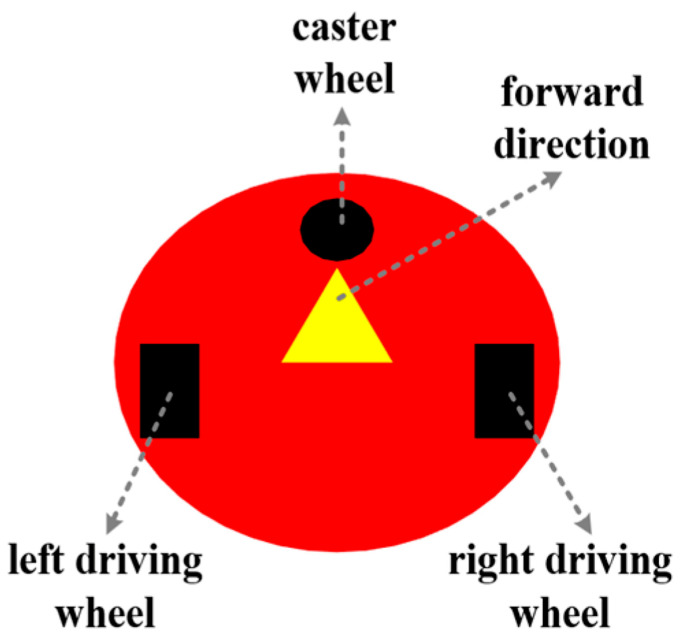
Mobile robot model.

The mobile robot used in the experiment adopts a two-wheeled differential drive structure [45], with a circular body design with a diameter of 0.8 m. Driving wheels are installed on both the left and right sides to provide motion power, and a universal wheel is added at the front to achieve auxiliary support functionality. The overall structure of the robot is shown in Figure 11.

### 5.3. Single Dynamic Obstacle Scenario (20 × 20)

This experiment constructs two sets of 20 × 20 grid maps for scenarios with a single dynamic obstacle. The Start point of both scenarios is uniformly set to (1, 2), and the end point to (18, 18), with the moving speed of the dynamic obstacle configured as 0.2 m/s. The core difference between the two scenarios lies in the distribution pattern of static obstacles, as well as the initial positions and motion trajectories of dynamic obstacles, which is designed to test the adaptability and robustness of the proposed EA-DFA under different obstacle constraint conditions.

• Simulation scenario 1

**Figure 12:**
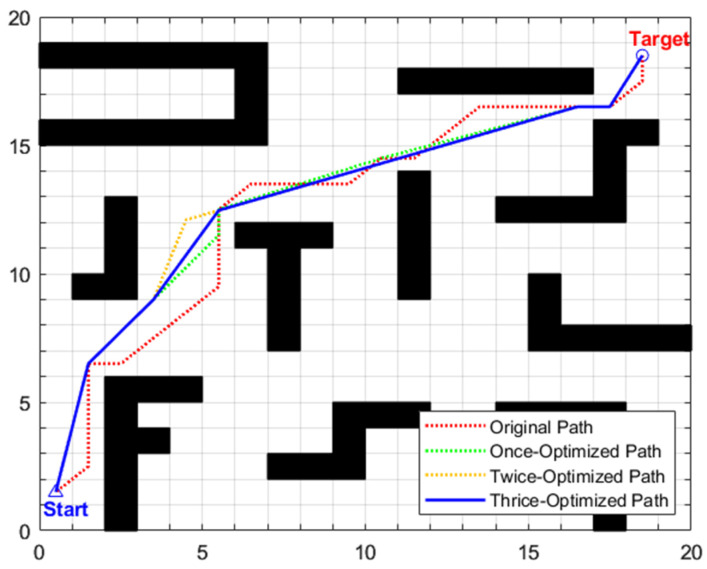
Global path optimization results of enhanced A* algorithm (20 × 20 map, scenario 1).

The global path planning results of scenario 1 are illustrated in Figure 12 and Table 7. From the perspective of computation time, the traditional A algorithm, without path optimization, has a search time of only 0.0811 s but with poor path quality. After introducing three-stage optimization, the total computation time of the enhanced A algorithm increases to 1.1175 s, with a total increment of 1.0364 s. Among them, the removal of redundant points in the first stage is the main source of time consumption: time increases from 0.0811 s to 1.0971 s, with an increment of 1.016 s, accounting for about 98% of the total additional time consumption. This is due to the need to traverse 25 initial intermediate transition points, perform collinearity judgment on triplet node groups one by one, and then perform obstacle distance detection on non-collinear node groups, resulting in intensive computation. The forward path relaxation in the second stage only increases the time by 0.0132 s, accounting for about 1.27%; the reverse path relaxation in the third stage increases the time by 0.0072 s, accounting for about 0.69%. The time consumption of path relaxation in the two stages is relatively low because only the six key intermediate transition points retained in the first stage are operated on, significantly reducing the number of nodes and computational complexity.

From the perspective of planning path quality, the primary optimization stage eliminates redundant nodes by leveraging a combined mechanism of collinearity judgment and safe distance detection, reducing the original path length from 29.7279 to 28.0479 and decreasing the number of intermediate transition points from 25 to 6. Subsequent second and third-stage optimizations adopt forward and reverse interpolation methods, respectively, selecting optimal transition points at key turning positions from disparate directions to straighten the path significantly. As depicted in Figure 12, the thrice-optimized path exhibits an “almost inflection-free” characteristic, with remarkably improved smoothness. The path length is further reduced to 27.2550, corresponding to a cumulative reduction of 8.32% relative to the original path, while the turn frequency and turning angle are notably diminished, thus enabling exceptionally smooth steering.

This smooth path profile can effectively reduce the energy consumption caused by frequent start–stop of the robot’s motor, while avoiding motion oscillations due to sharp turns, ensuring the stability of the robot during actual movement, and reducing the difficulty of low-level motion control. Although the path search time increased from 0.0811 s to 1.1175 s after three stages of optimization, the global path planning adopts an offline pre-computation mode, which consumes much less time than the actual motion time of the robot. Furthermore, based on the maximum linear velocity of 0.8 m/s set in the experiment, the optimized path length of 2.4729 m is shortened, which can save the robot approximately 3.1 s in actual motion. In summary, the path generated by the enhanced A* algorithm performs well in terms of path optimality, motion smoothness, and time efficiency, thereby providing a high-quality global reference path for subsequent local dynamic obstacle avoidance tasks.

**Figure 13:**
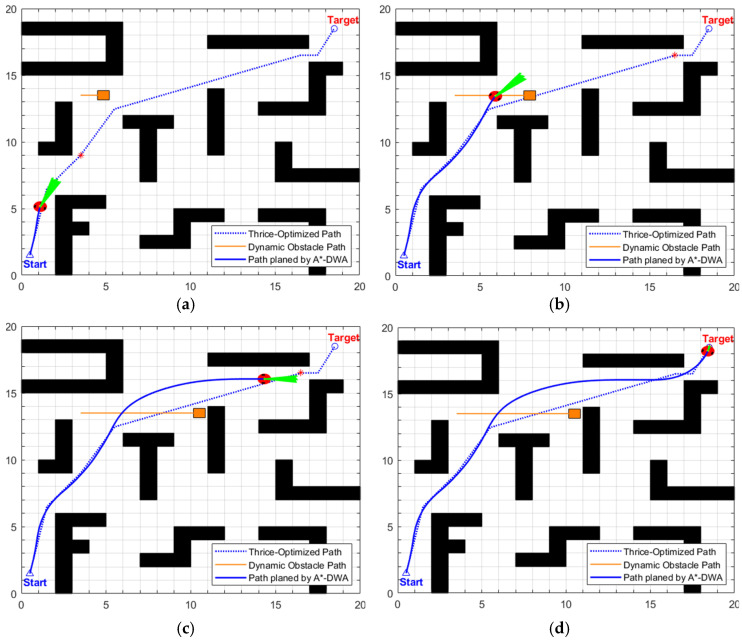
Dynamic path planning results of EA-DFA (20 × 20 map, scenario 1): (**a**–**d**) Snapshots illustrating different stages of the path planning process.

Based on the above static global path optimization results, further comparative verification experiments on dynamic path planning were conducted, using the proposed EA-DFA and the traditional DWA, respectively. The specific experimental results are analyzed as follows:

The dynamic path planned by the proposed EA-DFA is shown in Figure 13. Guided by the optimized static global path, the algorithm selects the intermediate transition points in the global path that are immediately adjacent to the robot’s current position as the real-time target points for DWA local planning. As can be observed from the path evolution process in Figure 13a–d, when a dynamic obstacle approaches the global path, the DWA local planning module rapidly generates a collision-free obstacle avoidance trajectory by sampling candidate trajectories and calculating the trajectory evaluation function in real time. Once obstacle avoidance is completed, the robot immediately returns to the global path without excessive detouring. Experimental data indicates that the dynamic path length planned by the proposed EA-DFA is 27.8460 m, with a planning time of 92.6340 s. The entire path satisfies safety distance constraints and poses no collision risk, which fully demonstrates the collaborative advantage of stepwise guidance by the global path and dynamic adjustment by local planning.

**Figure 14:**
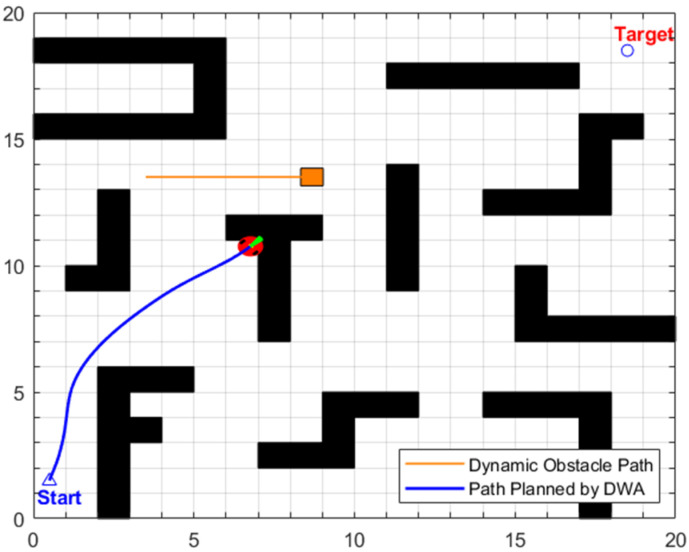
Dynamic path planning results of traditional DWA (20 × 20 map, scenario 1).

The dynamic path planned by the traditional DWA is shown in Figure 14. Due to the lack of stepwise and directional guidance provided by the global path, this algorithm consistently takes the global final target as the sole target point for local planning. Under the dual constraints of dynamic and static obstacles, the local feasible region is severely compressed. This method fails to search for a valid obstacle avoidance direction that satisfies both kinematic constraints and safety distance requirements simultaneously, eventually leading to corner deadlock and thus culminating in the failure of this dynamic path planning task.

In conclusion, the proposed EA-DFA, by employing the stepwise and directional guidance of intermediate transition points along the global path, effectively overcomes the critical drawback of the traditional DWA—a tendency to be susceptible to corner deadlocks. This approach provides a more dependable path planning solution for the efficient and stable navigation of mobile robots in dynamic environments.

• Simulation scenario 2

**Figure 15:**
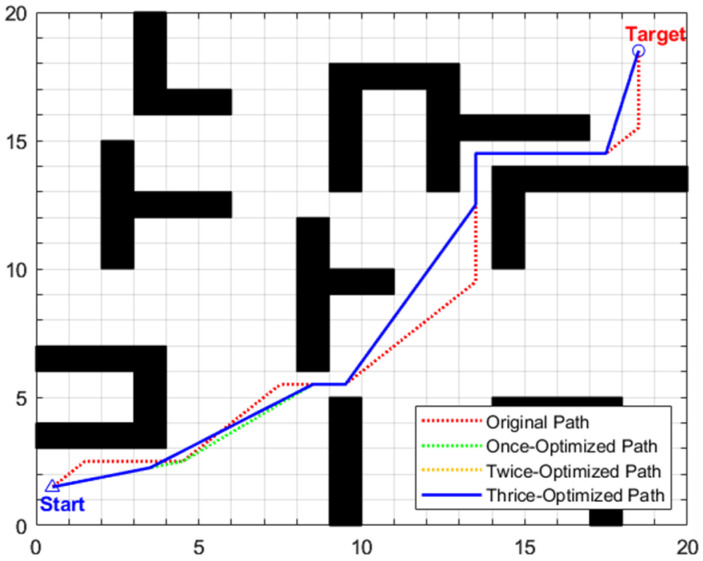
Global path optimization results of enhanced A* algorithm (20 × 20 map, scenario 2).

The global path planning results of scenario 2 are illustrated in Figure 15 and Table 8. For the traditional A* algorithm, the original path length is 29.7279 m, with 25 intermediate transition points and a search time of 0.0877 s. After three-stage optimization by the enhanced A* algorithm, the path length is reduced to 28.2411 m (a cumulative reduction of 5.00%), and the number of intermediate transition points is decreased to 6. Although the search time increases to 1.1431 s, according to the maximum linear velocity of 0.8 m/s set in the experiment, the shortened path length of 1.4868 m can save the robot approximately 1.9 s in actual motion. As depicted in Figure 15, the path after three-stage optimization exhibits distinct characteristics of “fewer inflection points and higher straightness” with smoother steering, which not only further enhances the stability and motion smoothness of robot navigation but also provides a continuous and reliable global reference path for subsequent local obstacle avoidance tasks in dynamic scenarios.

**Figure 16:**
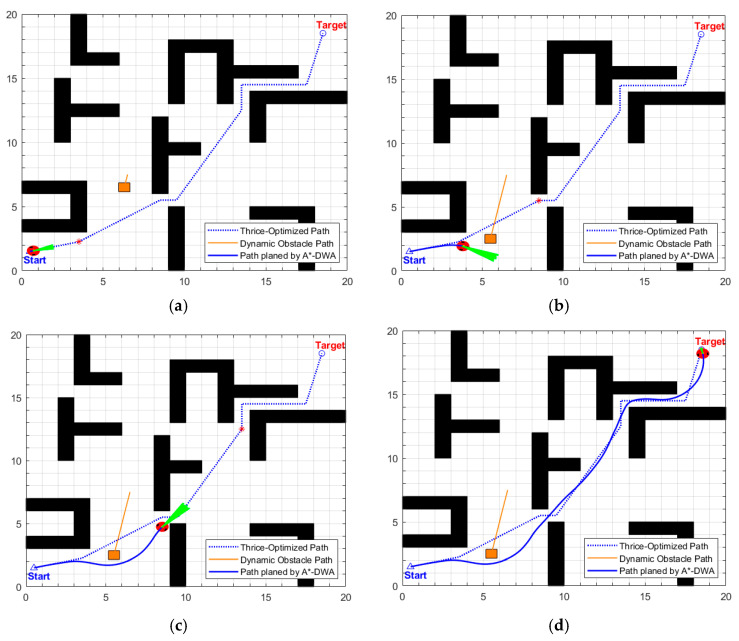
Dynamic path planning results of EA-DFA (20 × 20 map, scenario 2): (**a**–**d**) Snapshots illustrating different stages of the path planning process.

The dynamic path planning results of the proposed EA-DFA in scenario 2 are illustrated in Figure 16a–d. Taking the optimized global path as guidance, the algorithm generates obstacle avoidance trajectories and exhibits a core feature of notably enhanced degree of alignment between the motion trajectory and the global path, with no redundant detours. Experimental results demonstrate that its dynamic path length is 28.0520 m, with a planning time of 104.2640 s.

**Figure 17:**
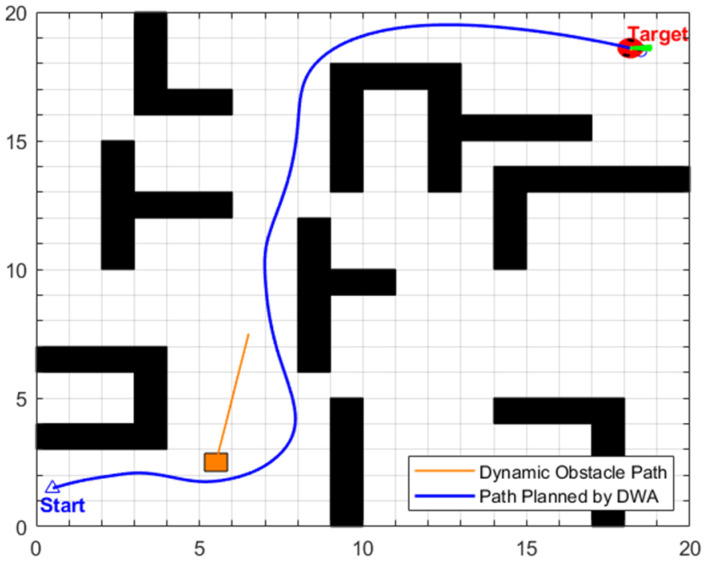
Dynamic path planning results of traditional DWA (20 × 20 map, scenario 2).

The planning results of the traditional DWA algorithm are illustrated in Figure 17. Although the latter can generate a complete path from the Start point to the end point, its path length reaches 33.4722 m, which is 19.32% longer than that of the proposed EA-DFA. Furthermore, due to the lack of global path guidance, the path exhibits a high degree of redundancy and frequent steering maneuvers, which not only increases the robot’s energy consumption and motion oscillations but also results in significantly lower navigation efficiency compared to the proposed EA-DFA. Notably, the planning time of the traditional DWA is 92.4290 s, 11.835 s shorter than that of the proposed EA-DFA. This discrepancy stems from the traditional DWA’s adoption of fixed safety evaluation weight (β_0_ = 0.1 in Table 6), which enables high-speed progression throughout the planning process. In contrast, the proposed EA-DFA adaptively adjusts the safety evaluation weights via Equation (9) in the high obstacle density areas of scenario 2, enhancing obstacle avoidance reliability by moderately reducing the local motion speed, which inevitably leads to an increase in planning time.

However, in terms of performance trade-off, the EA-DFA achieves a 19.32% reduction in path length (a decrease of 5.4202 m) at the cost of a modest additional 11.835 s in planning time. Its trajectory exhibits a high degree of alignment with the global path and smoother steering maneuvers, which not only effectively avoids the excessive energy consumption caused by the redundant paths of the traditional DWA but also provides more reliable collision avoidance assurance for navigation in high-obstacle areas. In contrast, the traditional DWA, despite incurring no collision risk and featuring a shorter planning time, exhibits a substantial increase in path length and extremely poor consistency with the global path. Such a short planning time performance, achieved at the expense of path optimality and trajectory adaptability, is difficult to meet the practical requirements of navigation accuracy and efficiency in complex dynamic scenarios. In summary, the proposed EA-DFA achieves a better balance among safety reliability, path optimality, and motion smoothness.

### 5.4. Multiple Dynamic Obstacles Scenario (30 × 30)

To further verify the comprehensive performance of the proposed EA-DFA in larger-scale maps and complex scenarios with multiple dynamic obstacles, this section constructs two sets of 30 × 30 grid environment experiments: scenario 1 is the alternating motion scenario of dual dynamic obstacles, and scenario 2 is a composite scenario of cross-moving obstacles and narrow corridors with variable widths. These two sets of experiments are designed to test the adaptability and robustness of the proposed algorithm under more complex dynamic obstacle constraints.

• Simulation scenario 1

In this scenario, two dynamic obstacles with different moving speeds are configured in the 30 × 30 grid map: the first dynamic obstacle moves at 0.4 m/s, and the second at 0.3 m/s.

**Figure 18:**
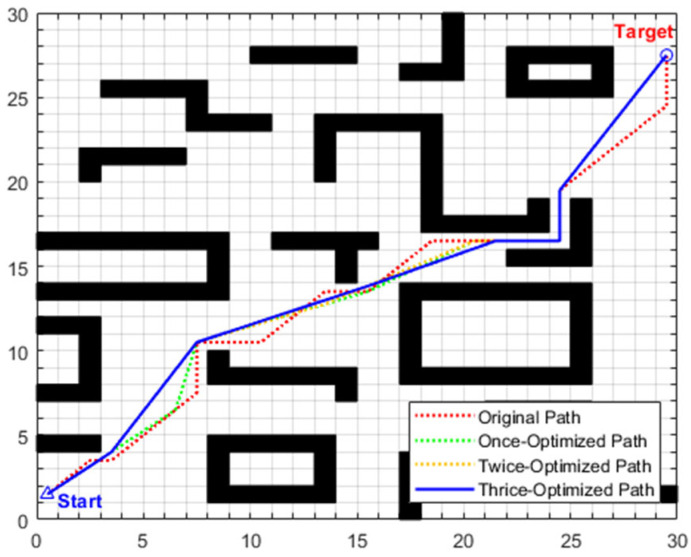
Global path optimization results of enhanced A* algorithm (30 × 30 map, scenario 1).

The global path planning results under this scenario are shown in Figure 18 and Table 9. The original path length of traditional A* is 45.0416 m, with 37 intermediate transition points and a search time of 0.1091 s; after three-stage optimization, the path length is reduced to 42.2041 (a cumulative reduction of 6.30%), and the intermediate transition points are reduced to 6. While the search time increases to 1.1539 s. This time increment mainly stems from the removal of redundant points in the first stage. Furthermore, a comparison between maps of different sizes shows that the computational time of the improved A* for the 30 × 30 map in this scenario only increases by 0.0364 s compared to the 20 × 20 map (scenario 1), indicating that the computational time of the improved algorithm has good robustness to map sizes. This characteristic is attributed to the fact that the core operations of the three-stage optimization all have linear time complexity and do not grow exponentially with map size, which not only ensures the optimization effect in this scenario but also endows the algorithm with practicality in larger and more complex scenarios. Additionally, although the planning time consumption has increased, based on the maximum linear velocity of 0.8 m/s set in the experiment, the optimized path can save approximately 3.55 s of motion time, which fully offsets the additional computational overhead.

**Figure 19:**
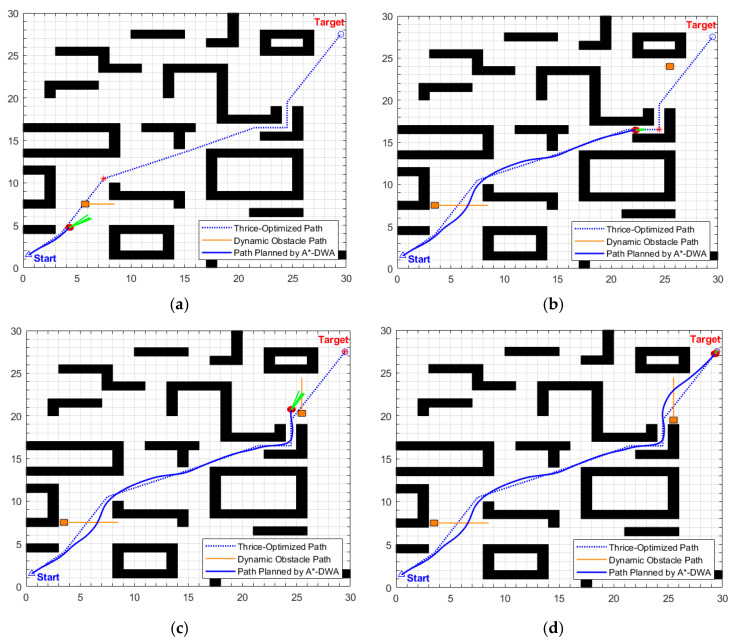
Dynamic path planning results of EA-DFA (30 × 30 map, scenario 1): (**a**–**d**) Snapshots illustrating different stages of the path planning process.

The dynamic path planning results of the proposed EA-DFA are illustrated in Figure 19a–d. Guided by the optimized global path, in the scenario of two alternately moving dynamic obstacles, its obstacle avoidance trajectories exhibit a high degree of alignment with the global path without excessive detours, and there is no collision risk with static or dynamic obstacles throughout the entire process. Experimental data demonstrates that the length of its dynamic path is 42.7830 m, with a planning time of 215.5020 s.

**Figure 20:**
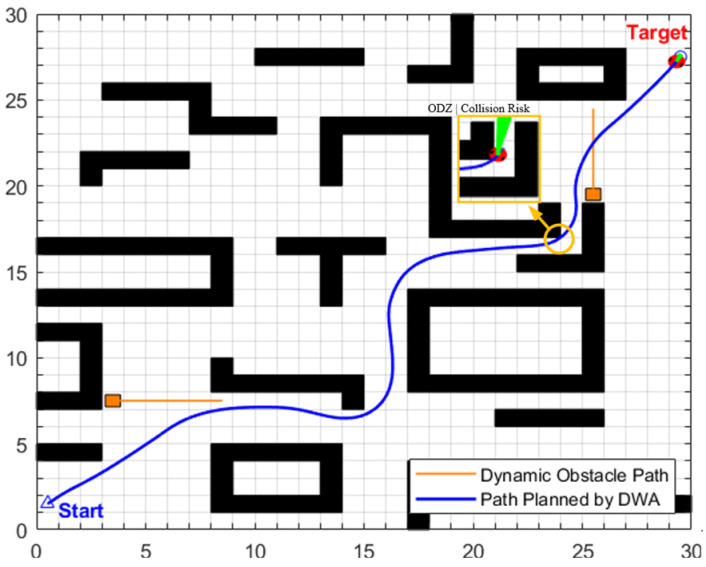
Dynamic path planning results of traditional DWA (30 × 30 map, scenario 1).

The planning results of the traditional DWA algorithm are illustrated in Figure 20. Although the planning task is completed, there are significant safety hazards in its planned path. As can be seen from the turning points in the path shown in Figure 20, the motion trajectory collides significantly with static obstacles. In terms of data, its path length is 45.1550 m (an increase of 5.55% compared to EA-DFA), and the planning time is 231.1678 s (an increase of 7.27% compared to EA-DFA). Due to the lack of global path guidance, the traditional DWA needs to frequently adjust its motion trajectory to avoid the interleaved interference of dynamic obstacles. This not only results in path redundancy and increased time consumption but also, more critically, as it adopts a fixed safety evaluation weight (β_0_ = 0.1), it fails to dynamically adapt to complex environmental, ultimately generating an unsafe path.

• Simulation scenario 2

This scenario constructs a composite environment of cross-moving obstacles and narrow corridors with variable widths on a 30 × 30 grid map. The specific settings are as follows: First, configure two cross-moving obstacles with a speed of 0.2 m/s, whose trajectories intersect the globally optimal path; second, set up two dynamic obstacles moving towards each other at 0.4 m/s, which will eventually stop on the globally optimal path; in addition, four dynamic obstacles (with a side length of 0.8 m) are arranged within a narrow corridor with an initial width of 2 m. These obstacles move along the direction of the corridor extension at a speed of 0.4 m/s, dynamically adjusting the passable width of the corridor to a range of 1.2 m to 2 m.

**Figure 21:**
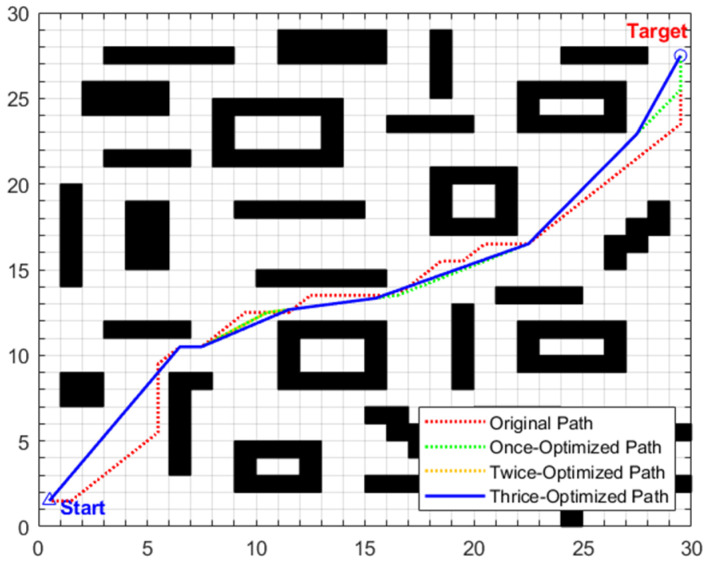
Global path optimization results of enhanced A* algorithm (30 × 30 map, scenario 2).

The global path optimization results under this composite scenario are shown in Figure 21 and Table 10. The original path generated by the traditional A* algorithm has a length of 44.4558 m, with 37 intermediate transition points and a search time of 0.0745 s. After three-stage optimization by the enhanced A* algorithm, the path length is reduced to 41.2378 m (a cumulative reduction of 7.23%), and the number of intermediate transition points is streamlined to 7. Considering the maximum linear velocity of 0.8 m/s set in the experiment, the optimized path can save approximately 4.02 s of actual motion time.

**Figure 22:**
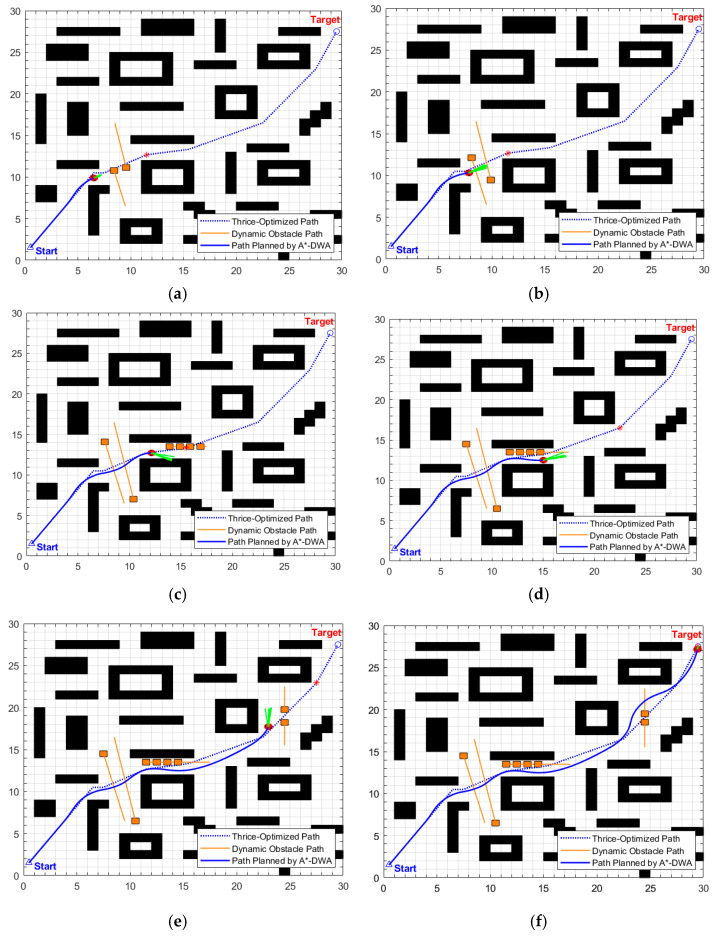
Dynamic path planning results of EA-DFA (30 × 30 map, scenario 2): (**a**–**f**) Snapshots illustrating different stages of the path planning process.

**Figure 23:**
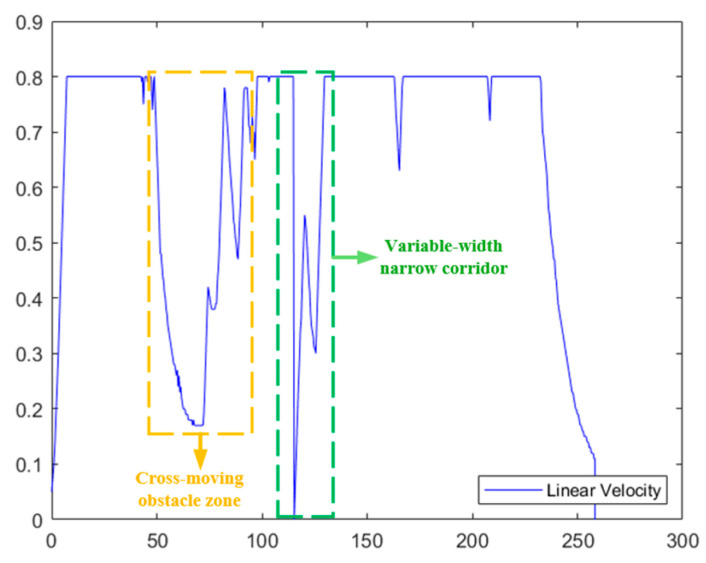
Linear velocity curve of mobile robot based on EA-DFA (30 × 30 map, scenario 2).

The key process of dynamic path planning for the proposed EA-DFA in scenario 2 is illustrated in Figure 22a–f: In Figure 22a, when the robot encounters cross-moving obstacles, the algorithm perceives increased risk through real-time calculation of the obstacle occupancy rate in the local environment, dynamically increases the weight of the safety evaluation term, and the relative proportion of the velocity evaluation term decreases accordingly. The algorithm prioritizes selecting low-velocity trajectories that meet safety constraints, and, as shown in the cross-moving obstacle zone of Figure 23, the robot’s traveling speed is significantly reduced. After the obstacles cross and separate and the environmental risk is alleviated, the weight of the safety evaluation term returns to β_0_, and the relative proportion of the velocity evaluation term rebounds, enabling the robot to accelerate smoothly. Upon entering the 2 m wide narrow corridors with variable widths shown in Figure 22c, as oncoming dynamic obstacles gradually approach the robot, the algorithm takes the globally optimal path as a directional anchor to correct the local traveling direction, while triggering the adaptive safety evaluation weight adjustment strategy again. By dynamically adjusting the weights to optimize speed decisions, the robot maintains a low speed and achieves precise obstacle avoidance in the complex area of the narrow corridors with variable widths. In the stage illustrated in Figure 22e, when the robot encounters two dynamic obstacles moving towards each other that eventually stop on the globally optimal path, the algorithm leverages the macro perspective advantage of the global path to autonomously determine the detour direction and plan a detour trajectory that fits the globally optimal path. Throughout the entire planning process, the proposed EA-DFA ultimately generates a planned path with a length of 41.8550 m, incurs a planning time of 258.3620 s, and exhibits no collision risk throughout.

**Figure 24:**
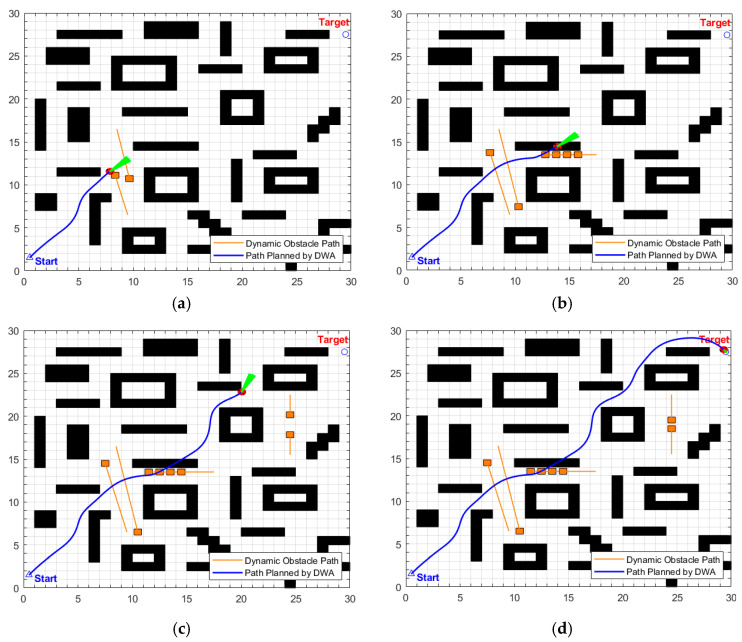
Dynamic path planning results of traditional DWA (30 × 30 map, scenario 2). (**a**–**d**) Snapshots illustrating different stages of the path planning process.

**Figure 25:**
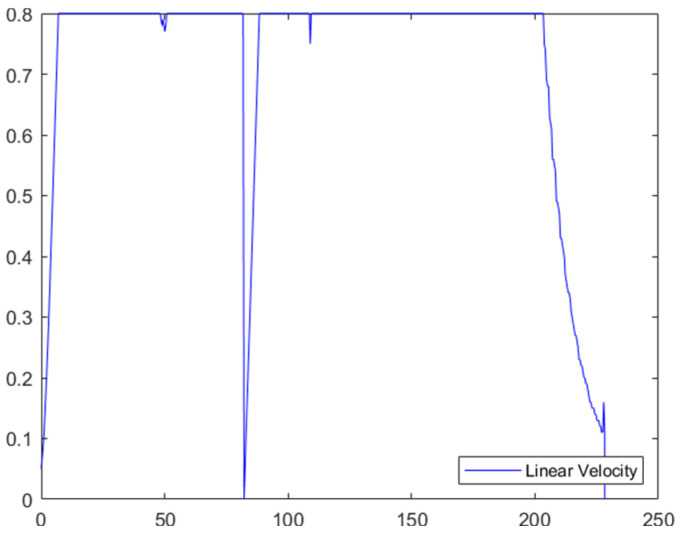
Linear velocity curve of mobile robot based on traditional DWA (30 × 30 map, scenario 2).

The planning results of the traditional DWA algorithm are illustrated in Figure 24a–d. Due to the lack of directional guidance from the global path and the adoption of fixed safety evaluation weights, it exhibits significant limitations in this complex scenario. In Figure 24a, when encountering cross-moving obstacles, the algorithm fails to correctly predict collision risks and directly collides with the cross-moving obstacles; after entering the narrow corridors, as it consistently takes the final target as the sole guide, it makes an incorrect decision on the obstacle avoidance direction and thus not only collides with oncoming dynamic obstacles but also crosses the static obstacle area; in Figure 24c, the algorithm collides with static obstacles again during the subsequent path search process, further confirming the blindness of its obstacle avoidance strategy. Combined with Figure 25, it can be observed that the traditional DWA maintains a relatively high traveling speed throughout the entire planning process and does not dynamically adjust speed decisions for high-risk areas, forming a sharp contrast with the risk adaptability of EA-DFA. In terms of data, the traditional DWA achieves a planned path length of 43.6803 m with a planning time of 228.2370 s, and multiple collisions with dynamic and static obstacles occur throughout the process. In contrast, relying on the synergistic support of enhanced global path guidance and adaptive dynamic adjustment of safety evaluation weights, the proposed EA-DFA realizes a shorter path and collision-free navigation, exhibiting superior safety, reliability, and environmental adaptability in complex dynamic scenarios.

In this section, we systematically verify the comprehensive performance of the proposed EA-DFA in the MATLAB simulation environment by constructing two sizes of grid maps (20 × 20 and 30 × 30) and setting up differentiated experimental scenarios including single dynamic obstacles and multiple dynamic obstacles. The results show that in environments of different scales and complexities, the proposed method performs well in terms of path length, path smoothness, and path safety. Which can provide a reliable simulation basis for the deployment of the algorithm in practical systems.

## 6. Experimental Validation with a Mobile Robot

**Figure 26:**
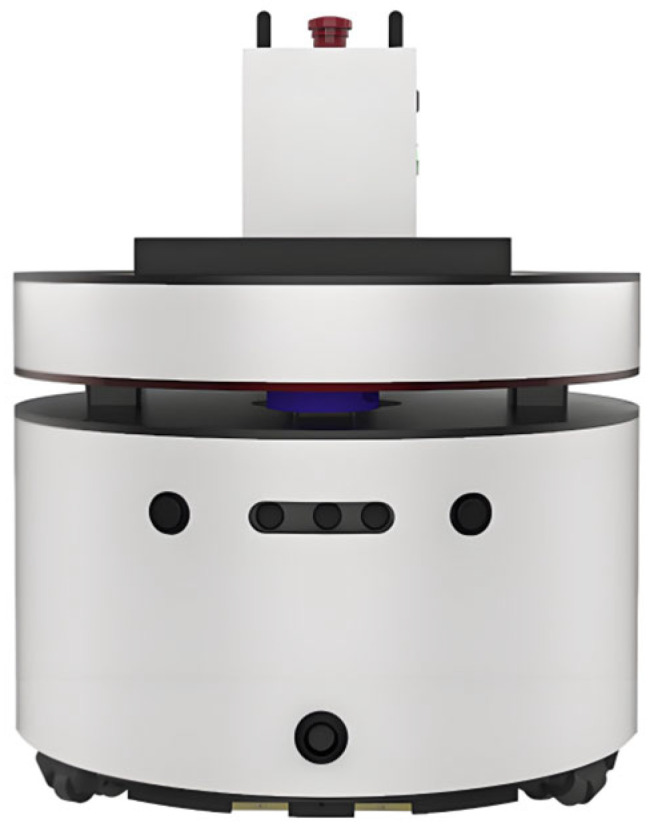
Physical prototype of the mobile robot chassis.

Considering that there are still some differences between the MATLAB simulation scenario and the actual physical system, in order to further verify the effectiveness and robustness of our Enhanced A*-DWA Fusion Algorithm in real scenarios, we built a physical experimental platform based on the ROS framework [46]. The mobile robot chassis used in the experiment is shown in Figure 26, which is equipped with perception devices such as two-dimensional LiDAR [47], depth camera, and ultrasonic sensor. The mobile robot used in this study was manufactured by Shenzhen Yuanchuangxing Technology Co., Ltd., located in Shenzhen, China.The software system is deployed on Ubuntu 20.04 with the ROS framework.

**Figure 27:**
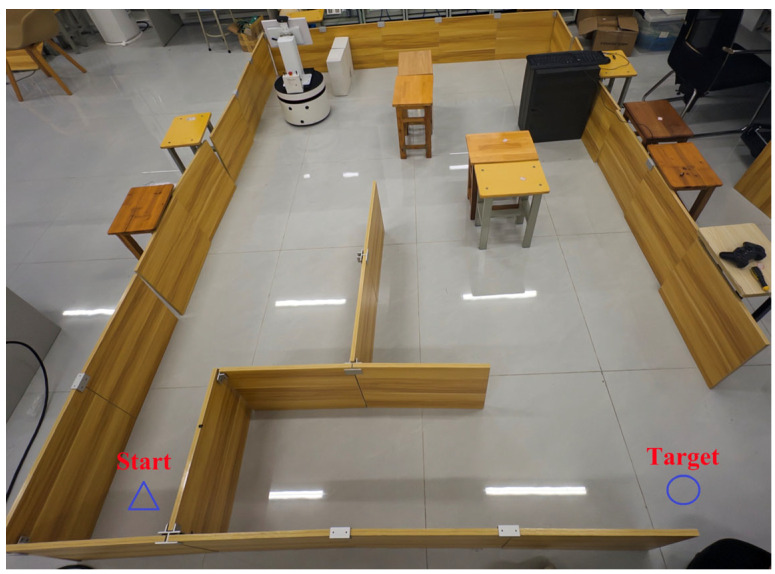
Layout of indoor experimental scene.

**Figure 28:**
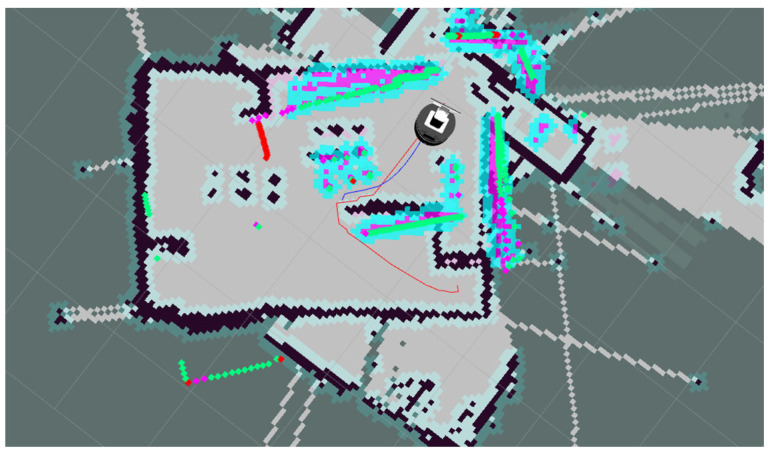
SLAM mapping visualization in Rviz.

The experiment was conducted in an indoor semi-structured environment, with the scene layout shown in Figure 27. The site size is approximately 5 m × 3 m, and there are static obstacles such as tables, chairs, and cardboard boxes arranged inside. Mobile robots use 2D LiDAR and depth cameras to collect real environmental data, generate a grid map of the experimental environment via the open-source SLAM algorithm Gmapping [48], and then the ROS navigation module superimposed traversal cost information on this grid map to finally generate a costmap for path planning [49]. Figure 28 shows the constructed map observed in Rviz software (version 1.14.13), where the black area represents obstacles, the gray area represents feasible areas, the red line is the path planned by the robot globally, the blue line is the adjusted path during local obstacle avoidance, the green area marks the map boundary, and the remaining colored areas are visual representations of the costmap, where the brighter the color, the higher the cost of passage, and the robot will actively avoid the corresponding areas.

The experiment was divided into two groups, both of which were conducted in the same indoor structured venue with the same starting and target points, as shown in Figure 27. And use the proposed EA-DFA in this article for online planning.

• Experiment 1: Static environment navigation

**Figure 29:**
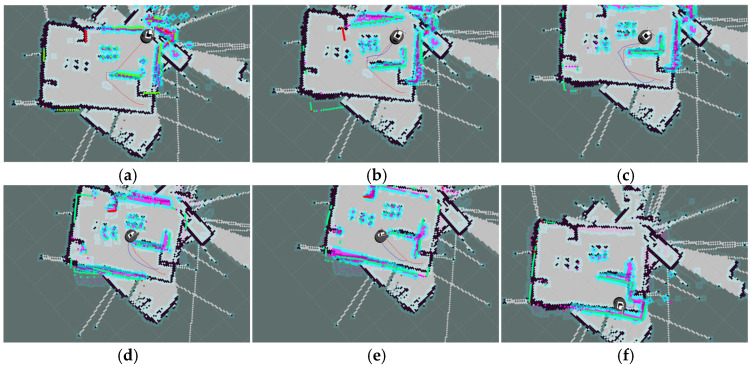
Sequence diagram of the SLAM mapping process (experiment 1). (**a**–**f**) Snapshots illustrating different stages of the path planning process.

**Figure 30:**
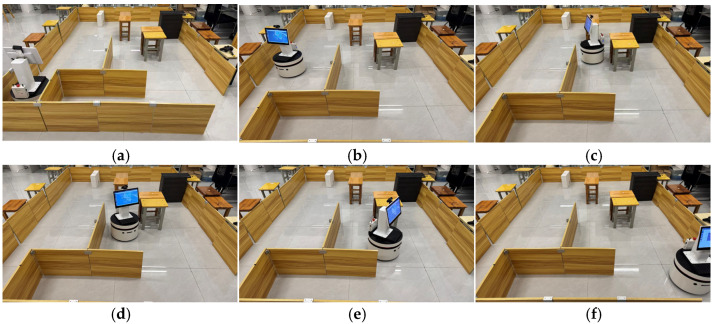
Sequence diagram of robot movement in the real scene (experiment 1). (**a**–**f**) Snapshots illustrating different stages of the path planning process.

The main purpose of this experiment is to verify the global path generated by the enhanced A * algorithm and its synergistic effect with the improved DWA local planning algorithm in scenarios without introducing dynamic obstacles. The SLAM mapping process and the actual robot movement process are shown in Figure 29 and Figure 30, respectively. The results show that the robot can smoothly travel along the global path generated by the enhanced A * algorithm from the starting point (red curve in the figure). The improved DWA algorithm dynamically adjusts the trajectory based on real-time sensor data (blue curve), and the generated local trajectory fits well with the global path. There were no collisions during the entire process, and the trajectory smoothness was good, verifying the feasibility and stability of the algorithm in static environments. There were no collisions during the entire process, and the trajectory exhibited high smoothness, which verifies the feasibility and stability of the algorithm proposed in this paper in static environments.

• Experiment 2: Dynamic environment navigation

**Figure 31:**
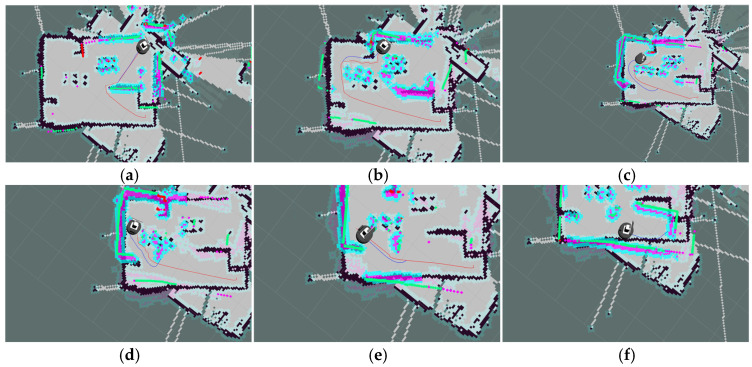
Sequence diagram of the SLAM mapping process (experiment 2). (**a**–**f**) Snapshots illustrating different stages of the path planning process.

**Figure 32:**
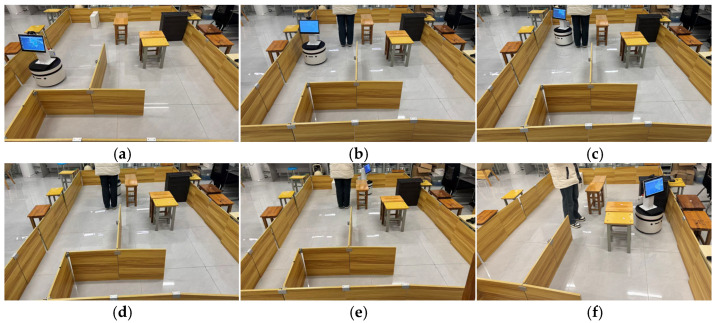
Sequence diagram of robot movement in the real scene (experiment 2): (**a**–**f**) Snapshots illustrating different stages of the path planning process.

In this group of experiments, pedestrian was introduced as dynamic obstacles during the mobile robot’s movement to verify the real-time obstacle avoidance and path replanning capabilities of the proposed EA-DFA in dynamic environments. When pedestrian enter the robot’s perception range and approach its globally planned path, the robot detects in real time the reduction in obstacle distance via 2D LiDAR and then triggers the local planner to enter obstacle avoidance mode. As shown in Figure 31, during the actual movement process, the robot perceives that the original global path is obstructed when approaching pedestrian (Figure 31b–d), and the local planner guides it to detour safely. The corresponding SLAM mapping process is shown in Figure 32. It can be seen that the robot’s motion was continuous throughout the obstacle avoidance process without any pauses, demonstrating excellent dynamic response and real-time planning performance.

In this section, we conducted physical verification of the proposed EA-DFA based on the ROS mobile robot platform in an actual indoor environment. Through two groups of experiments, the results demonstrate that: in static scenarios, the algorithm can achieve smooth tracking of the global path with a high degree of fitting; In dynamic scenarios, algorithm can provide real-time and safe obstacle avoidance responses to sudden obstacles and quickly return to the global path direction after obstacle avoidance. The physical experiment verified the effectiveness and robustness of the algorithm under real sensor data, environmental noise, and motion control, which provides an experimental basis for its application in practical mobile robot systems.

## 7. Conclusion and Future Work

### 7.1. Summary

This paper focuses on the path planning problem of mobile robots in dynamic and complex environments, and conducted a systematic study covering algorithm improvement, integration, and physical verification. The main work and conclusions are as follows:

(1) A three-stage optimization strategy for improving the A* algorithm was proposed. By removing redundant points, performing forward and reverse path relaxation on the initial path generated by the traditional A* algorithm, the inherent problems of frequent turns and poor smoothness of the original path are effectively addressed, yielding a high-quality global reference path that better meets the robot’s motion tracking requirements.

(2) Two key enhancement measures were proposed for the DWA. By utilizing optimized global path information to set a precise initial heading angle, the problem of directional blindness in the initial stage of traditional methods was resolved. Through constructing an evaluation function integrating dynamic obstacle perception, a safety distance saturation mechanism, and adaptive weight adjustment, the safety, guidance, and environmental adaptability of local planning in dynamic environments are significantly improved.

(3) A fusion planning framework featuring deep collaboration between global and local planning was constructed. Taking the global path generated by the enhanced A* algorithm as macroscopic guidance, the system selects the intermediate transition point ahead as the local goal in real-time to drive the improved DWA for decision-making and obstacle avoidance within a rolling window. This design theoretically ensures the unification of global optimality and local real-time performance.

(4) The effectiveness of the algorithm was comprehensively verified through simulation and physical experiments. In the simulation phase, the improved fusion algorithm demonstrates superior path length, smoothness, and success rate in various static and dynamic scenarios. More importantly, physical verification based on the ROS-based mobile robot platform shows that the algorithm can handle real sensor data, environmental noise, and motion control constraints, exhibiting excellent robustness, real-time performance, and practicality in both static navigation and dynamic obstacle avoidance tasks. This completes the key closed loop from theoretical simulation to practical application.

### 7.2. Future Work

Although the algorithm proposed in this paper has achieved the expected results in both simulation and physical experiments, there is still room for further deepening and expansion of the research. Future work can focus on the following two aspects:

(1) Enhancement of Dynamic Environment Modeling and Prediction Capabilities: The handling of dynamic obstacles in this paper primarily relies on real-time ranging information. In the future, multimodal perception data such as visual data can be integrated, and deep learning-based trajectory prediction algorithms can be introduced to achieve early and accurate prediction of the motion intentions and trajectories of dynamic obstacles, thereby generating more forward-looking and strategic obstacle avoidance paths.

(2) Extension towards Complex Multi-Task and Multi-Robot Systems: Current research focuses on point-to-point navigation for single robots. In the future, this fusion framework can be extended to multi-robot collaborative work scenarios, and in-depth research can be conducted on system-level collaborative planning methods including dynamic task allocation, path coordination optimization, and real-time conflict resolution, so as to improve the overall system efficiency and robustness in complex operation scenarios.

## Figures and Tables

**Table 1:** Performance comparison of A*-DWA fusion algorithms.

Algorithm	Global Planning Optimization	Local Planning Optimization	Adaptability to Environmental Complexity	Experimental Validation	Core Advantages
SOA-DWA (Ref. [25])	path pruning + B-spline smoothing	added the distance evaluation between the reference trajectory and the global path, the path direction evaluation, and the dynamic obstacle avoidance evaluation	fixed weights, non-responsive to environmental complexity	MATLAB simulation + physical verification	optimal path smoothness, high global path alignment
improved A*-DWA (Ref. [26])	heuristic function optimization (obstacle rate introduced) + redundant node elimination + child node expansion optimization (safety distance constrained)	added distance evaluation for unknown obstacles	fixed weights, non-responsive to environmental complexity	MATLAB simulation	high search efficiency, safe and reliable planned paths
ADA-DWA (Ref. [27])	dynamic hybrid heuristic function + key-node selection strategy + direction-aware neighborhood Search	adaptive weight adjustment (speed, heading angle, obstacle distance, target orientation)	proposes an adaptive weight adjustment strategy, but lacks a quantitative link to environmental complexity	MATLAB simulation + physical verification	efficient path planning, robust dynamic obstacle avoidance, strong environmental adaptability
EA-DFA (this paper)	three-stage hierarchical optimization: redundant point reduction + bidirectional relaxation	initial heading angle calibration + adaptive evaluation function (dynamic perception, safety distance saturation, environment-adaptive weight)	proposes an adaptive weight adjustment strategy, and establishes an explicit, quantitative link to environmental complexity	MATLAB simulation + physical verification	safe and efficient path planning, intelligent and flexible obstacle avoidance, suitable for complex scenarios

**Table 2:** Pseudocode for Once-Optimization.

BEGIN
n←2
A←path[1],B←path [2],C←path [3]
WHILE(n < length(path))
IF collinear (A, B, C)
Keep A invariant
Delete B from Path
ELSE
h←minDistanceToObstacles (A, C, Map)
IF h < Safe_Distance
A←path [n]
ELSE
Keep A invariant
Delete B from Path
END IF
END IF
n←n+1▷ Move to next window
B←path [n], C←path [n+1]
END WHILE
Return path
END BEGIN

**Table 3:** Pseudocode for Twice-Optimization.

BEGIN
i←1
WHILE (i <length(path1)-2)
A←Path1 [i], B←Path1 [i+1], C←Path1 [i+2]
Q← interpolate(A, B, σ) ▷ Generate Candidate points
FOR j ← 1 to size(Q)
Candidate ← Q [j]
h←minDistanceToObstacles(A,C,Candidate,Map)
IF h>Safe_Distance
Path1 [i+1]←Candidate
break
End IF
End FOR
i←i +1 ▷ Move to next window
END WHILE
Return Path1
END BEGIN

**Table 4:** Performance comparison under different safety distance saturation thresholds.

Safety Distance Saturation Threshold	Path Length (m)	Time (s)	Planning Status
0.5⋅Safe_Distance	17.0221	37.4450	Unsafe, frequent collisions
1⋅Safe_Distance	17.5284	27.0140	Safe but low planning efficiency
2⋅Safe_Distance	18.1070	23.2600	Optimal balance of safety and efficiency
3⋅Safe_Distance	19.8601	21.9870	Limited efficiency improvement, increased path length
4⋅Safe_Distance	23.4629	18.2190	Excessive detour, reduced global optimization
5⋅Safe_Distance	>60 (Target not reached)	No valid path	Planning failed, local looping

**Table 5:** Performance comparison under different safety weight adjustment strategies.

Strategy	Path Length (m)	Time (s)	Average Velocity (m/s)
$\beta =\beta_{0}$	12.6145	23.5620	0.4435
$\beta =\beta_{0}\cdot (1+\rho )$	12.9096	25.5870	0.4209
$\beta =\beta_{0}\cdot 2\cdot (1+\rho )$	13.3239	29.6180	0.3825
$\beta =\beta_{0}\cdot 3\cdot (1+\rho )$	13.5089	39.8800	0.2745

**Table 6:** Parameter settings for the EA-DFA.

Parameter Category	Parameter Name	Value	Description
Mobile Robot Mechanical Performance Parameters	$v_{\max}$ (m/s)	0.8	Maximum Linear Velocity
	$v_{\min}$ (m/s)	0	Minimum Linear Velocity
	$\omega_{\max}$ (rad/s)	45.0/180 ×pi	Maximum Angular Velocity
	$\omega_{\min}$ (rad/s)	0	Minimum Angular Velocity
	$\overset{\cdot }{v_{a}}$ (m/s^2^)	0.5	Linear Acceleration
	$\overset{\cdot }{\omega_{a}}$ (rad/s^2^)	50.0/18 ×pi	Angular Acceleration
DWA Algorithm Basic Parameters	Safe_Distance (m)	0.75	Safety Distance
	$T_{sim}$(s)	3.0	The total simulation time
	Δt(s)	0.1	Sampling Period
Evaluation Function Weight Coefficients	α	0.05	Guidance Evaluation Weight
	$\beta_{0}$	0.1	Base Safety Evaluation Weight
	γ	0.2	Velocity Evaluation Weight

**Table 7:** Performance comparison of global path (20 × 20 map, scenario 1).

Algorithm	Path Length	Number of Intermediate Transition Points	Search Time
Original Path	29.7279	25	0.0811
Once-Optimized Path	28.0479	6	1.0971
Twice-Optimized Path	27.5752	6	1.1103
Thrice-Optimized Path	27.2550	6	1.1175

**Table 8:** Performance comparison of global path (20 × 20 map, scenario 2).

Algorithm	Path Length	Number of Intermediate Transition Points	Search Time
Original Path	29.7279	25	0.0877
Once-Optimized Path	28.3085	6	1.1165
Twice-Optimized Path	28.2411	6	1.1347
Thrice-Optimized Path	28.2411	6	1.1431

**Table 9:** Performance comparison of global path (30 × 30 map, scenario 1).

Algorithm	Path Length	Number of Intermediate Transition Points	Search Time
Original Path	45.0416	37	0.1091
Once-Optimized Path	42.7423	6	1.1315
Twice-Optimized Path	42.3205	6	1.1469
Thrice-Optimized Path	42.2041	6	1.1539

**Table 10:** Performance comparison of global path (30 × 30 map, scenario 2).

Algorithm	Path Length	Number of Intermediate Transition Points	Search Time
Original Path	44.4558	37	0.0745
Once-Optimized Path	41.6149	7	1.1001
Twice-Optimized Path	41.3080	7	1.1131
Thrice-Optimized Path	41.2378	7	1.1197

## Data Availability

The original contributions presented in this study are included in the article. Further inquiries can be directed to the corresponding author.
